# Supplementary material for: Inhibition of urease-mediated ammonia production by 2-octynohydroxamic acid in hepatic encephalopathy
Source: Nat Commun. 2024 Mar 12;15:2226. doi: 10.1038/s41467-024-46481-8 (PMC10933438; doi:10.1038/s41467-024-46481-8)
Supplement: Supplementary file 1 — Supplementary Information [file 41467_2024_46481_MOESM1_ESM.pdf]

Supplementary information

Inhibition of urease-mediated ammonia production by 2-octynohydroxamic acid in hepatic encephalopathy

Diana Evstafeva[1], Filip Ilievski[1], Yinyin Bao[1], Zhi Luo[1], Boris Abramovic[1], Sunghyun Kang[1], Christian Steuer[1], Elita Montanari[1], Tommaso Casalini[2], Dunja Simicic[3,4], Dario Sessa[5], Stefanita-Octavian Mitrea[3,4], Katarzyna Pierzchala[3,4], Cristina Cudalbu[3,4], Chelsie E. Armbruster[6], Jean-Christophe Leroux[1*]

[1]Institute of Pharmaceutical Sciences, Department of Chemistry and Applied Biosciences, ETH Zurich, Zurich, Switzerland

[2]Institute for Chemical and Bioengineering, Department of Chemistry and Applied Biosciences, ETH Zurich, Zurich, Switzerland

[3]CIBM Center for Biomedical Imaging, Lausanne, Switzerland

[4]Animal Imaging and Technology, EPFL, Lausanne, Switzerland

[5]Swiss Pediatric Liver Center, Department of Pediatrics, Gynecology and Obstetrics, University Hospitals Geneva and University of Geneva, Geneva, Switzerland

[6]Department of Microbiology and Immunology, Jacobs School of Medicine and Biomedical Sciences, State University of New York at Buffalo, Buffalo, New York, USA

E-mail: jleroux@ethz.ch

Table of Contents

Supplementary Figures 2
Supplementary Tables 40
Supplementary Methods 47
Supplementary References 56

Supplementary Figures

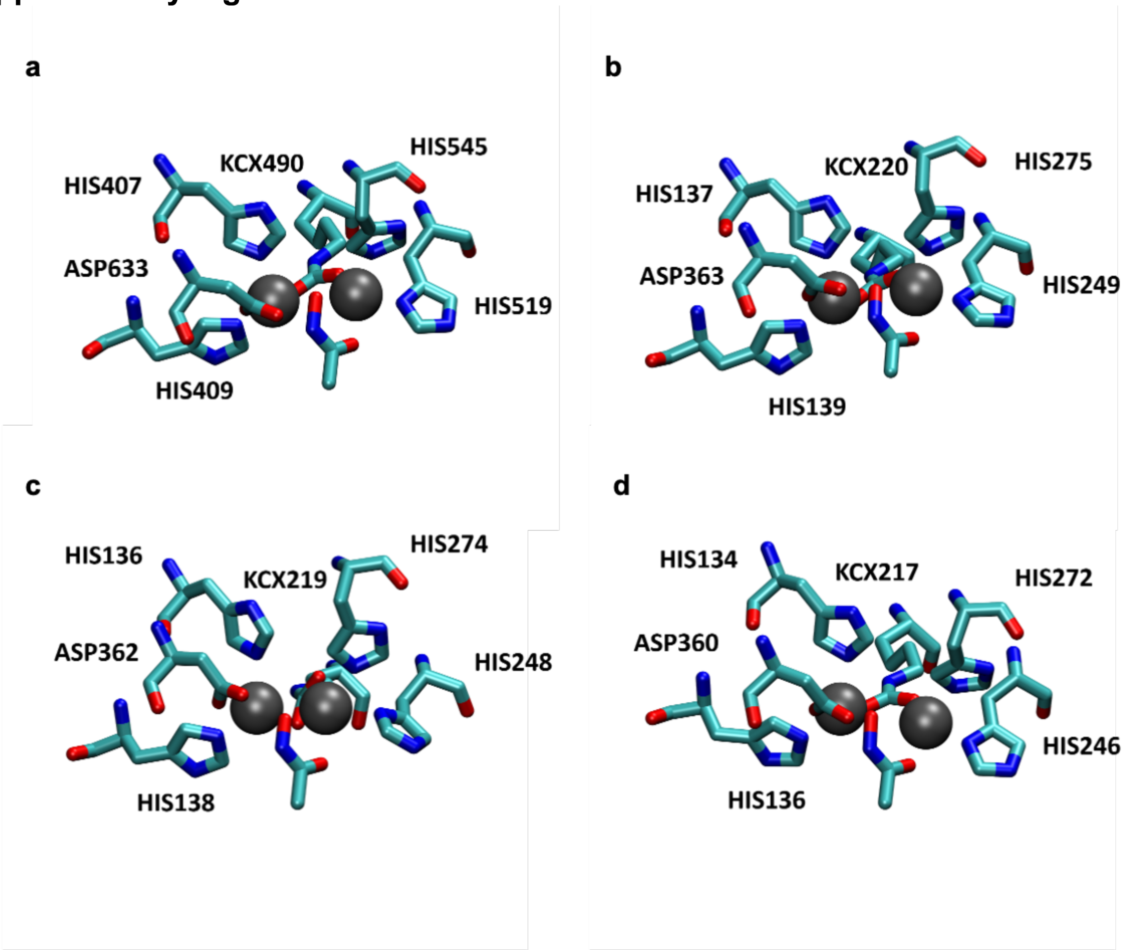

Supplementary Fig. 1. The binding pose of acetohydroxamic acid (AHA) in different ureases from the available crystallographic structures: (a) PDB ID: 4H9M[1]; (b) PDB ID: 4UBP[2,3]; (c) PDB ID: 1E9Y[4,5]; (d) PDB ID: 1FWE[6,7]. Hydrogen atoms were not included in the original structures and were not added. Amino acids included in the binding site and AHA are represented in the licorice style, nickel ions are represented as gray van der Waals spheres. The numbering of amino acids is given according to the respective structure. The structures were visualized using the Visual Molecular Dynamics (VMD) 1.9.3[8].

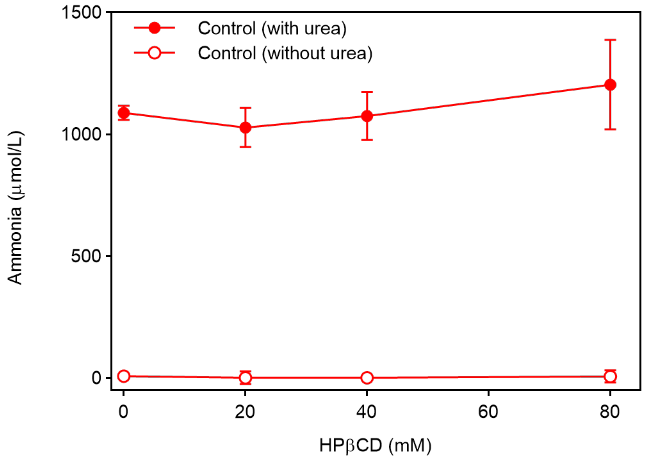

Supplementary Fig. 2. In vitro anti-ureolytic activity of (2-hydroxypropyl)-β-cyclodextrin (HPβCD). Concentrations of ammonia produced in caecal content after 30 min incubation with urea (n = 3) or without urea (n = 3) in the presence of increasing concentrations of HPβCD. Data are expressed as mean ± standard deviation (SD) from n experiments. Source data are provided as a Source data file.

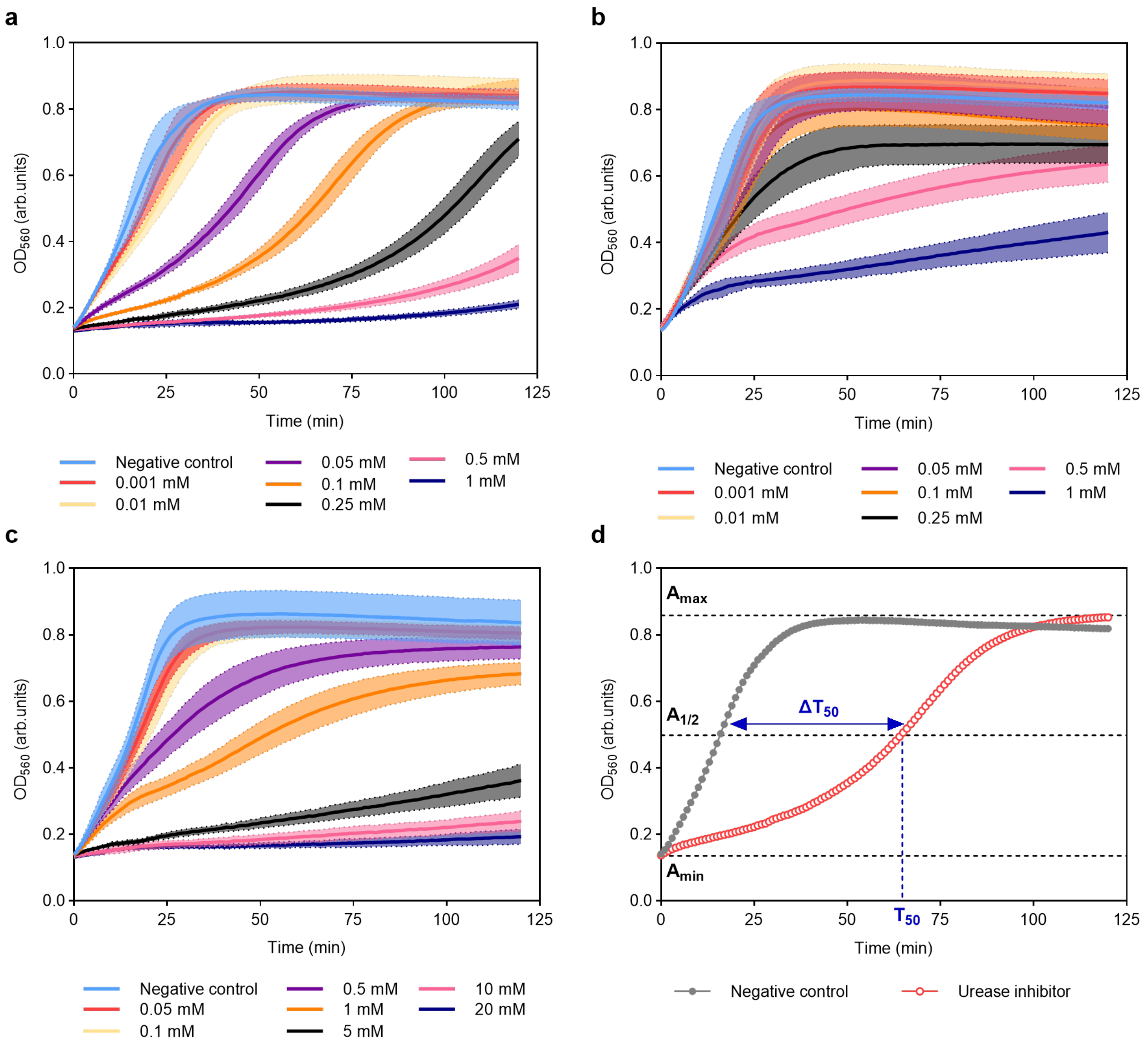

Supplementary Fig. 3. Jack bean urease activity in the presence of 2-octynohydroxamic acid (2-octynoHA), octanohydroxamic acid (OHA) and acetohydroxamic acid (AHA) in a pH-based assay with phenol red. Phenol red absorbance at 560 nm (OD_{560}) was measured in the samples containing urease and urea with increasing concentrations of **(a)** 2-octynoHA (*n* = 4), **(b)** OHA (*n* = 4) and **(c)** AHA (*n* = 3) every 60 s over 2 h at 37 °C. Data are represented as lines connecting mean values from *n* experiments, the area within standard deviation is shaded. **d** Schematic illustration of the assay. A mixture of Jack bean urease, urea and phenol red was incubated with a urease inhibitor or ultra-pure water (negative control) for 2 h at 37 °C. The delayed increase of OD_{560} values in the case of urease inhibitor indicates the delay in pH rise which is attributed to inhibited urea hydrolysis. The maximum absorbance (A_{max}), half of the maximum absorbance ($A_{1/2}$), minimum absorbance (A_{min}) values were identified for the negative control. The time (T_{50}) at which the absorbance value of the sample with a urease inhibitor reaches $A_{1/2}$ of the control sample was determined. Source data are provided as a Source data file.

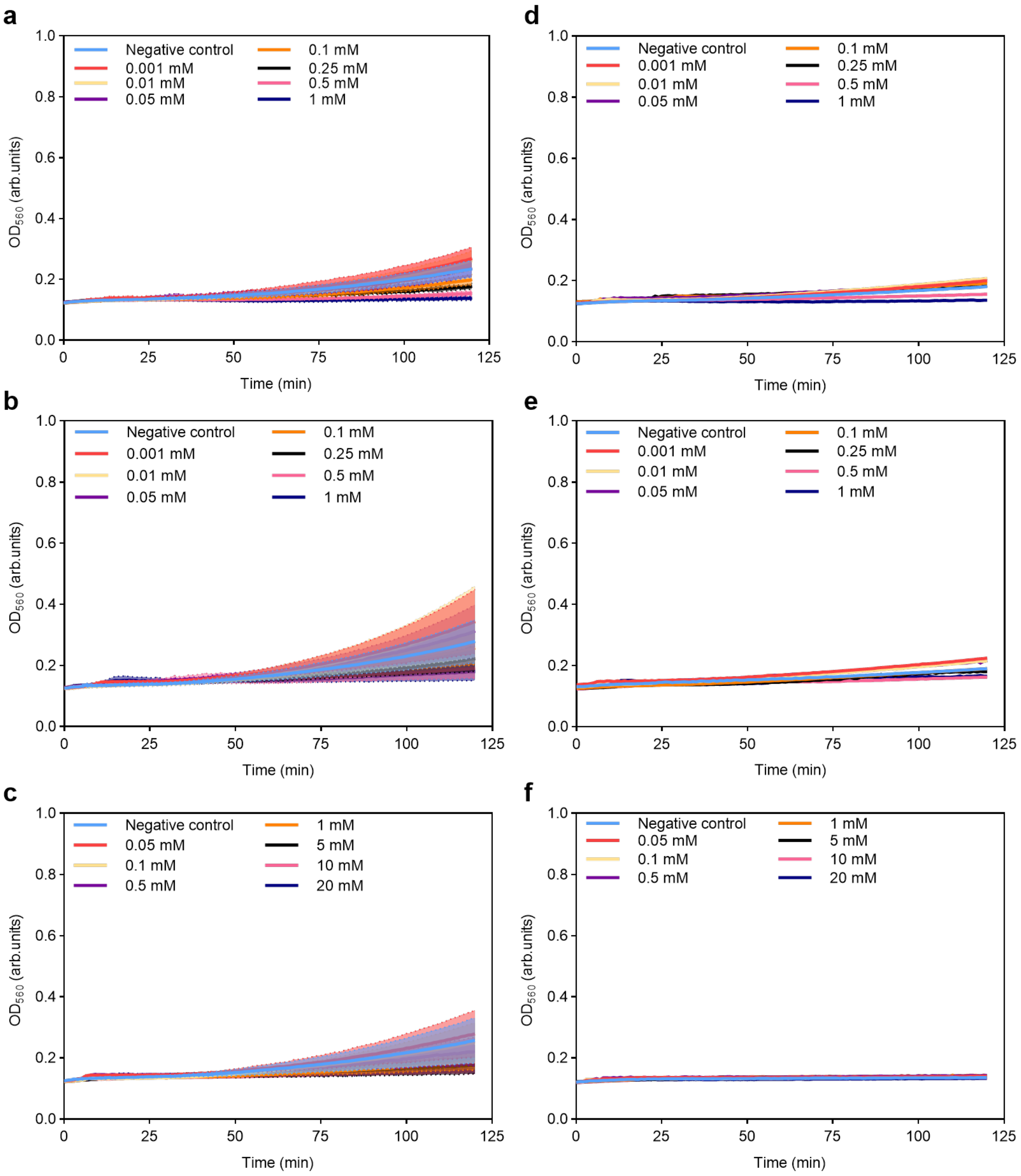

Supplementary Fig. 4. Evolution of phenol red absorbance at 560 nm (OD_{560}) in the presence of 2-octynohydroxamic acid (2-octynoHA), octanohydroxamic acid (OHA) and acetohydroxamic acid (AHA) with or without urea. a – c The OD_{560} values of control samples (without urease) containing urea and phenol red with increasing concentrations of **(a)** 2-octynoHA (*n* = 4), **(b)** OHA (*n* = 4) and **(c)** AHA (*n* = 3). The OD_{560} values slightly increase over 2 h incubation at 37°C possibly due to urea breakdown. Data are expressed as mean ± SD from *n* experiments. **d – f** The OD_{560} values of control samples (without urease and urea) containing phenol red with increasing concentrations of **(d)** 2-octynoHA, **(e)** OHA and **(f)** AHA. No change in OD_{560} of phenol red was observed. Data are expressed as mean from three technical replicates. Source data are provided as a Source data file.

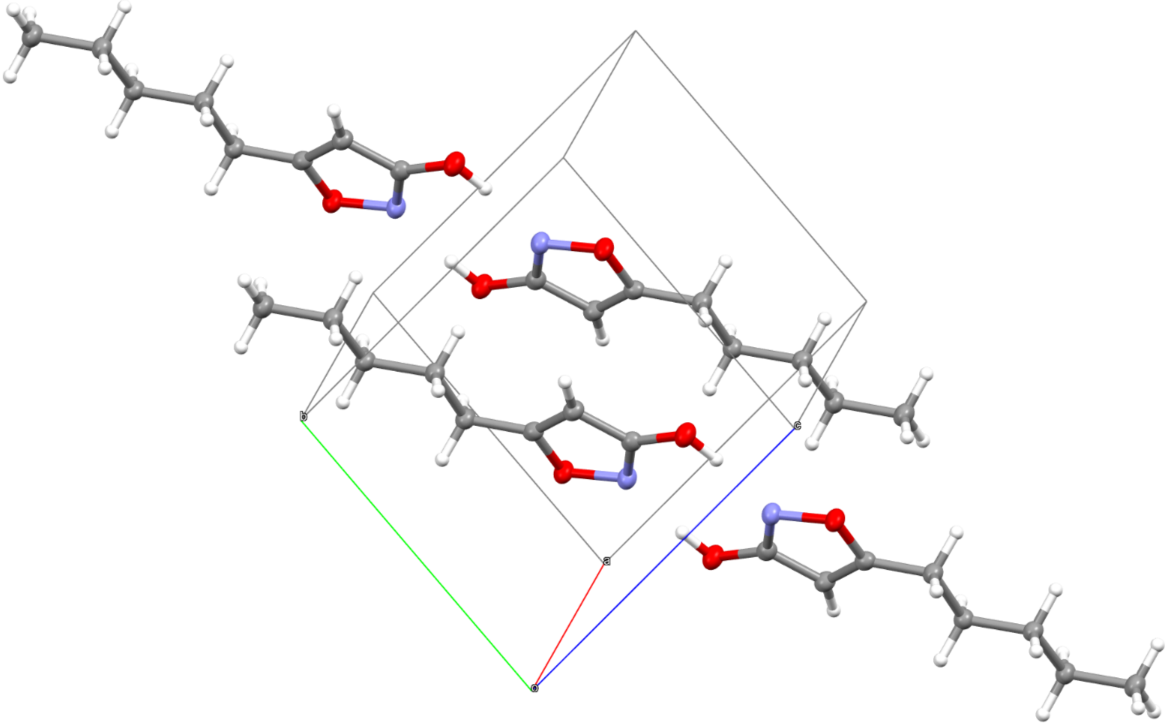

Supplementary Fig. 5. Crystal structure of 5-pentylisoxazol-3-ol showing O-H···N contacts. Ellipsoids are depicted at 50% probability; hydrogen atoms are shown as fixed-size spheres for clarity.

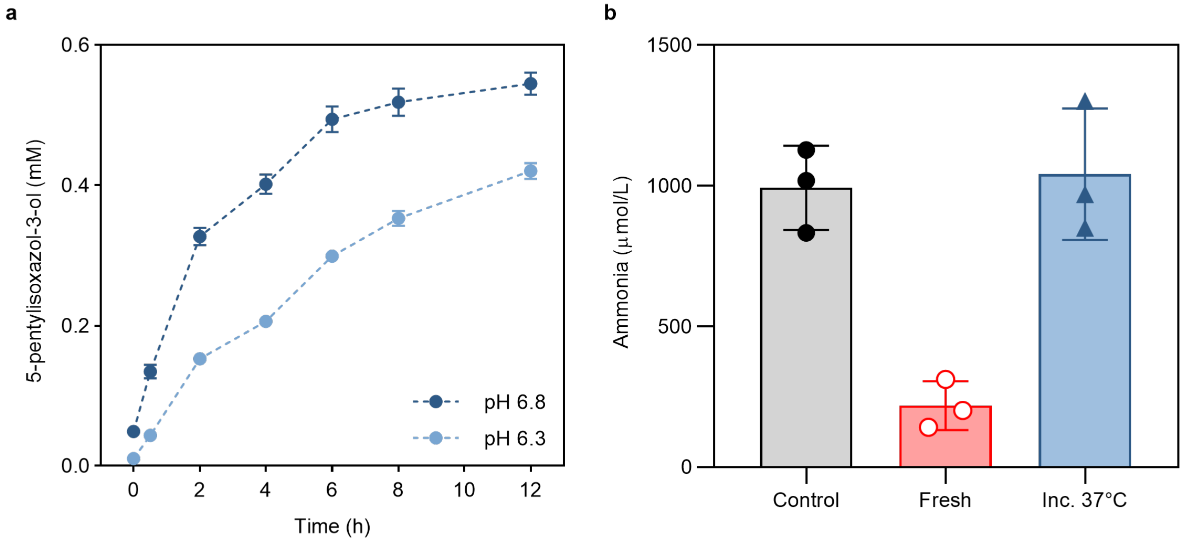

Supplementary Fig. 6. In vitro characterization of 5-pentylisoxazol-3-ol. a Formation of 5-pentylisoxazol-3-ol from 2-octynohydroxamic acid (2-octynoHA) over 12 h in phosphate buffer at pH 6.8 and at pH 6.3 (*n* = 3). **b** Concentrations of ammonia produced from urea hydrolysis in caecal content after 30 min incubation with freshly prepared solution of 0.25 mM 2-octynoHA (fresh) in phosphate buffer (pH 6.8) (*n* = 3), a solution of 0.25 mM 2-octynoHA incubated overnight at 37 °C (Inc. 37 °C, *n* = 3) or without an inhibitor (control, *n* = 3). No urease inhibitory activity was observed for overnight incubated solution of 2-octynoHA suggesting its conversion to 5-pentylisoxazol-3-ol which is not active against urease. All data are expressed as mean ± SD from *n* experiments. Source data are provided as a Source data file.

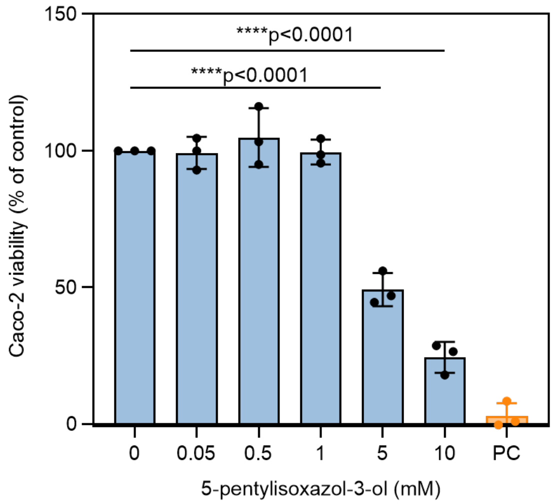

Supplementary Fig. 7. Cytotoxicity assessment of 5-pentylisoxazol-3-ol. Caco-2 cell viability in the presence of 5-pentylisoxazol-3-ol compared to the medium control (*n* = 3). Positive control (PC, 10 mM hydrogen peroxide) is presented as an orange bar (*n* = 3). The data are presented as mean ± SD from *n* experiments. Statistical significance was calculated by one-way analysis of variance (ANOVA) with Tukey's multiple comparisons test with ****$p < 0.0001$ vs. medium control. All p-values are reported in Supplementary Data 13. Source data are provided as a Source data file.

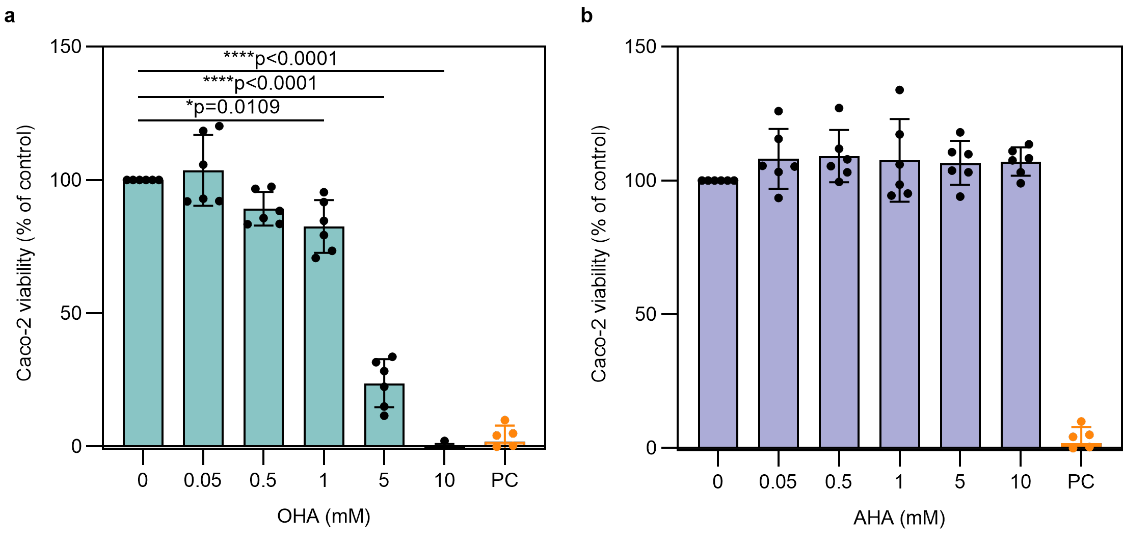

Supplementary Fig. 8. Cytotoxicity assessment of AHA and OHA. Caco-2 cell viability in the presence of **(a)** OHA and **(b)** AHA compared to the medium control (n = 6). Positive control (PC, 10 mM hydrogen peroxide) is presented as an orange bar (n = 6). Cytotoxicity experiments for 2-octynoHA, OHA and AHA were performed in parallel and therefore had the same positive control samples which are shown in these both figures and Fig. 3a in the Results section. The data are presented as mean ± SD from n experiments. Statistical significance was calculated by one-way ANOVA with Tukey's multiple comparisons test with * $p < 0.05$, **** $p < 0.0001$ vs. medium control. All p-values are reported in Supplementary Data 14-15. Source data are provided as a Source data file.

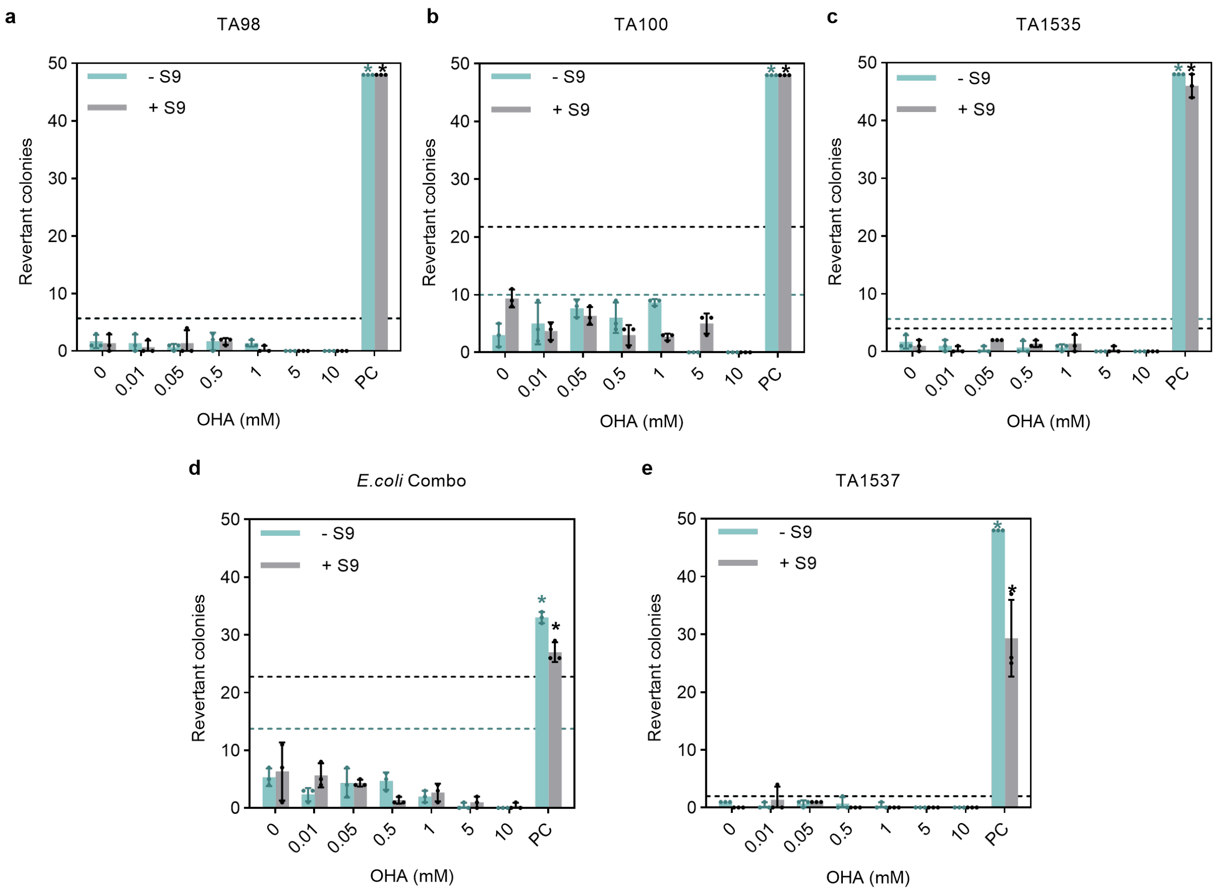

Supplementary Fig. 9. Mutagenicity assessment of octanohydroxamic acid (OHA). Mutagenicity of OHA in **(a)** TA98 (n = 3), **(b)** TA100 (n = 3), **(c)** TA1535 (n = 3), **(e)** TA1537 (n = 3) *S. typhimurium* strains and in **(d)** a combination of *E. coli* wp2 uvrA and *E. coli* wp2 [pKM101] strains (*E. coli* Combo, n = 3) is expressed as number of wells with revertant colonies per 48 wells. Mutagenicity was assessed with (+ S9) and without (- S9) liver homogenate (S9 fraction). The black and green dashed lines show a 2-fold increase over a baseline level for the experiments with and without S9 fraction, respectively, indicating the minimal number of revertant colonies per 48 wells at which the tested inhibitor's concentration can be considered mutagenic. Mutagenic test samples are indicated with black or green asterisk for the experiments with and without S9 fraction, respectively. Positive controls (PC) for each strain are listed in Supplementary Tables 4 and 5. Data are expressed as mean ± SD from n experiments. Source data are provided as a Source data file.

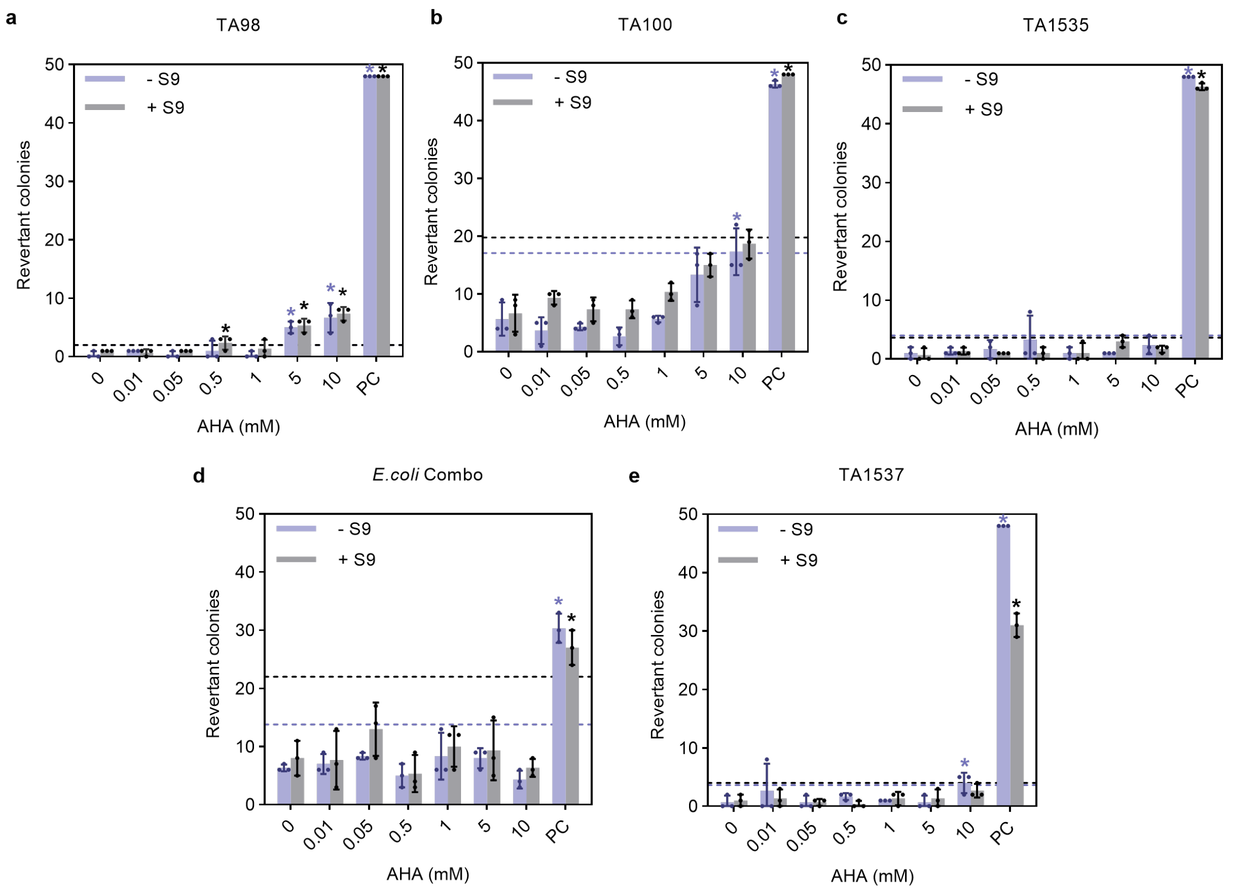

Supplementary Fig. 10. Mutagenicity assessment of acetohydroxamic acid (AHA). Mutagenicity of AHA in **(a)** TA98 (*n* = 3), **(b)** TA100 (*n* = 3), **(c)** TA1535 (*n* = 3), **(e)** TA1537 (*n* = 3) *S. typhimurium* strains and in **(d)** a combination of *E. coli* wp2 uvrA and *E. coli* wp2 [pKM101] strains (*E. coli* Combo, *n* = 3) is expressed as number of wells with revertant colonies per 48 wells. Mutagenicity was assessed with (+ S9) and without (- S9) liver homogenate (S9 fraction). The black and purple dashed lines show a 2-fold increase over a baseline level for the experiments with and without S9 fraction, respectively, indicating the minimal number of revertant colonies per 48 wells at which the tested inhibitor's concentration can be considered mutagenic. Mutagenic test samples are indicated with black or purple asterisk for the experiments with and without S9 fraction, respectively. Positive controls (PC) for each strain are listed in Supplementary Tables 4 and 5. Data are expressed as mean ± SD from *n* experiments. Source data are provided as a Source data file.

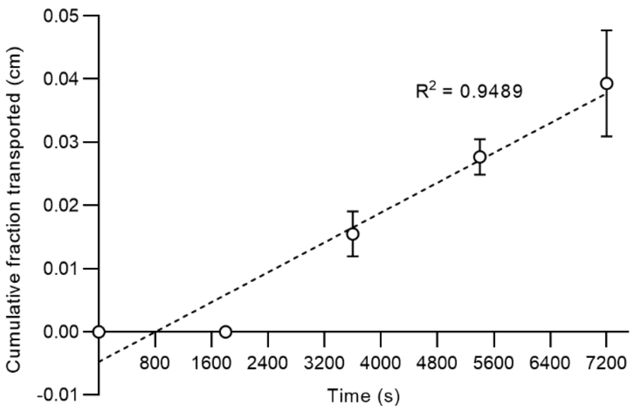

Supplementary Fig. 11. Permeability of 5-pentylisoxazol-3-ol across the Caco-2 monolayer. Cumulative fraction of 5-pentylisoxazol-3-ol transported across the Caco-2 monolayer over 2 h (7,200 s) (*n* = 3). Data are expressed as mean ± SD from *n* experiments. Source data are provided as a Source data file.

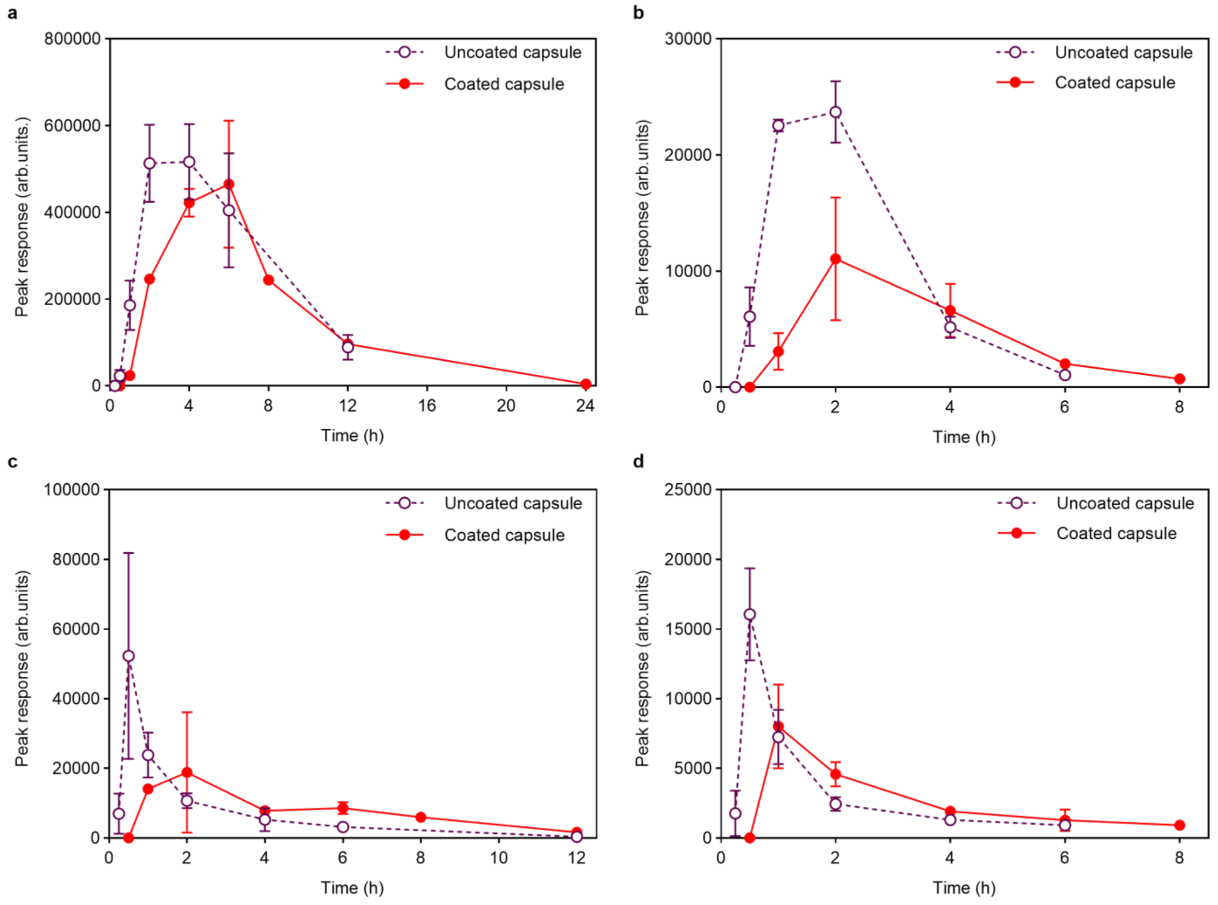

Supplementary Fig. 12. **Pharmacokinetic (PK) profiles of metabolites of 2-octynohydroxamic acid (2-octynoHA) in dogs following oral administration of 2-octynoHA in control uncoated or enteric capsules. a** Peak response profile of metabolite S12 (*n* = 3 for uncoated capsule, *n* = 2 for coated capsule). **b** Peak response profile of metabolite S22 (*n* = 3 for uncoated capsule, *n* = 2 for coated capsule). **c** Peak response profile of metabolite S32 (*n* = 3 at 0.25 – 6 h, *n* = 1 at 12 h for uncoated capsule; *n* = 2 for coated capsule). **d** Peak response profile of metabolite S33 (*n* = 3 at 0.25 – 2 h, *n* = 2 at 4 h, *n* = 1 at 6 h for uncoated capsule; *n* = 2 at 0.5 – 6 h, *n* = 1 at 8 h for coated capsule). Data are expressed as mean ± SD from *n* animals. Source data are provided as a Source data file.

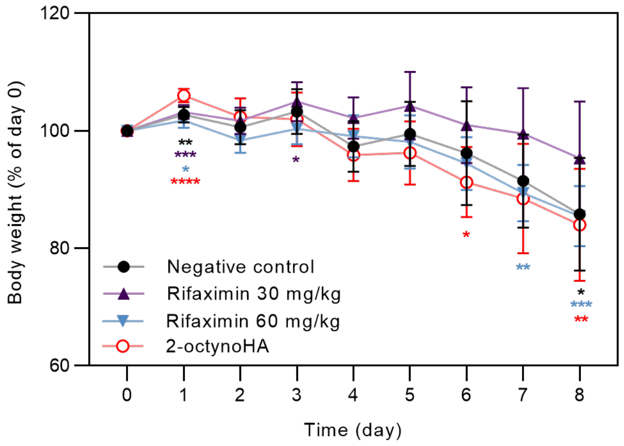

Supplementary Fig. 13. Evolution of the body weight in rats with acute liver injury induced by N-nitrosodiethylamine (DEN). Rats were treated with (2-hydroxypropyl)-β-cyclodextrin (HPβCD, negative control, *n* = 10), 15 mg/kg 2-octynoHA (in a solution with HPβCD, *n* = 10), 30 mg/kg (*n* = 10) or 60 mg/kg (*n* = 10) rifaximin. The negative control group and the groups receiving 2-octynoHA solution or 60 mg/kg rifaximin showed significant weight loss towards the end of the study (days 6 – 8) compared to day 0. Data are expressed as mean ± SD from *n* animals. Statistical significance within the group was calculated by one-way repeated measures ANOVA with Tukey's multiple comparisons test with * $p < 0.05$, ** $p < 0.01$, *** $p < 0.001$, **** $p < 0.0001$ vs. body weight on day 0. All p-values are reported in Supplementary Data 16. Source data are provided as a Source data file.

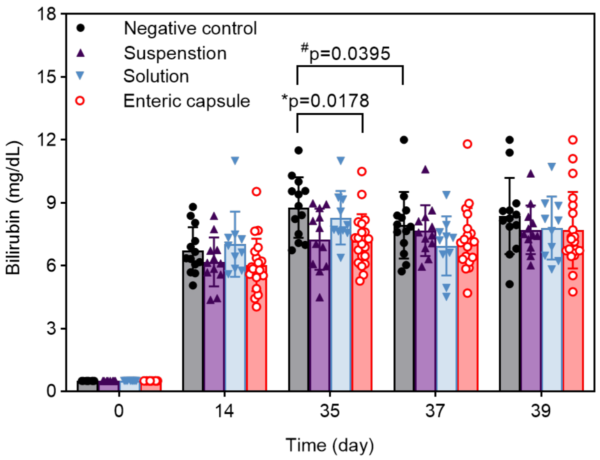

Supplementary Fig. 14. Plasma bilirubin levels in bile duct ligated (BDL) rats. Plasma bilirubin levels were below 0.5 mg/dL in all animals before the BDL surgery which was followed by a substantial increase already on day 14 post-surgery, confirming the development of liver disease. Data are expressed as mean ± SD from *n* animals where *n* = 13 for the negative control group, *n* = 12, *n* = 10, *n* = 20 for the groups receiving 2-octynohydroxamic acid (2-octynoHA) suspension, solution and enteric capsule, respectively. Statistical significance between groups during the treatment course (days 35-39) was calculated by two-way repeated measures ANOVA with Tukey's multiple comparisons test with *$p < 0.05$. Statistical significance between treatment days (days 35-39) within each group was calculated by one-

way repeated measures ANOVA with Tukey's multiple comparisons test with $^{\#}p < 0.05$ vs. day 35. All p-values are reported in Supplementary Data 17. Source data are provided as a Source data file.

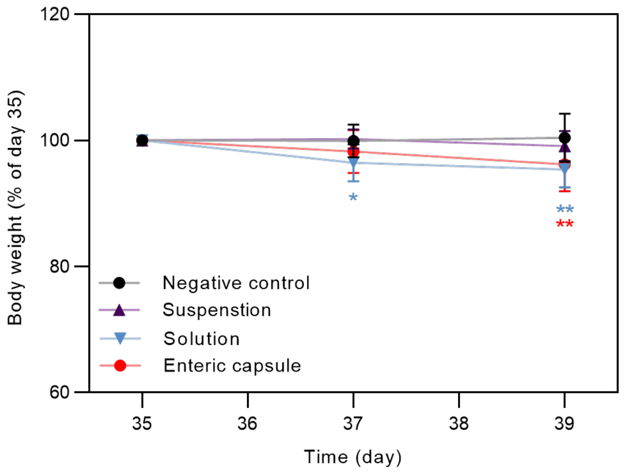

Supplementary Fig. 15. Body weight of BDL rats during the treatment with different formulations of 2-octynohydroxamic acid (2-octynoHA). Animals receiving 2-octynoHA formulated as a solution with (2-hydroxypropyl)-β-cyclodextrin (HPβCD, 30 mg/kg, twice a day) or as an enteric capsule (10 mg per rat, once a day) showed a statistically significant reduction of the body weight on days 37 and 39 post-surgery corresponding to the third and fifth days of the treatment, respectively. Data are expressed as mean ± SD from *n* animals where $n = 13$, $n = 12$, $n = 10$ and $n = 20$ for the negative control, 2-octynoHA suspension, 2-octynoHA solution and enteric capsule groups, respectively. Statistical significance between treatment days (days 35-39) within each group was calculated by one-way repeated measures ANOVA with Tukey's multiple comparisons test with $^{*}p < 0.05$, $^{**}p < 0.01$ vs. body weight on day 35. All p-values are reported in Supplementary Data 18. Source data are provided as a Source data file.

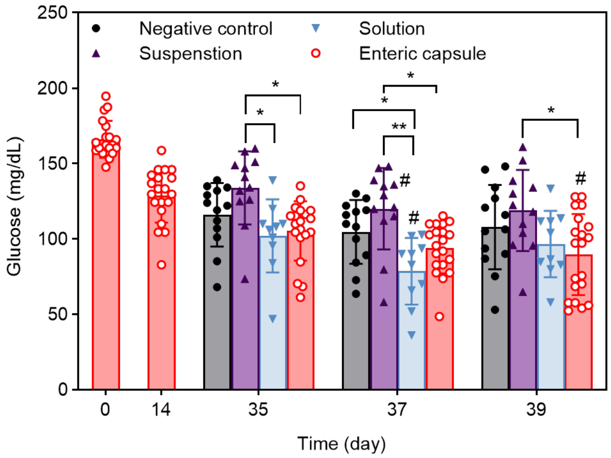

Supplementary Fig. 16. Plasma glucose levels in bile duct ligated (BDL) rats. Glucose measurements during the treatment (days 35 – 39) showed a slight decrease on day 37 vs. 35 in groups receiving suspension or solution of 2-octynohydroxamic acid (2-octynoHA), and on day 39 vs. 35 in the group receiving 2-octynoHA with the enteric capsule. The group treated with a solution of 2-octynoHA displayed significantly lower glucose levels compared to both the negative control and suspension groups on day 37. Data are expressed as mean ± SD from *n* animals where *n* = 13 for the negative control group; *n* = 12, *n* = 10, *n* = 20 for the groups receiving 2-octynoHA suspension, solution and enteric capsule, respectively. Statistical significance between groups during the treatment course (days 35-39) was calculated by two-way repeated measures ANOVA with Tukey's multiple comparisons test with $^{*}p < 0.05$, $^{**}p < 0.01$. Statistical significance between treatment days (days 35-39) within each group was calculated by one-way repeated measures ANOVA with Tukey's multiple comparisons test with $^{\#}p < 0.05$ vs. day 35. All p-values are reported in Supplementary Data 19. Source data are provided as a Source data file.

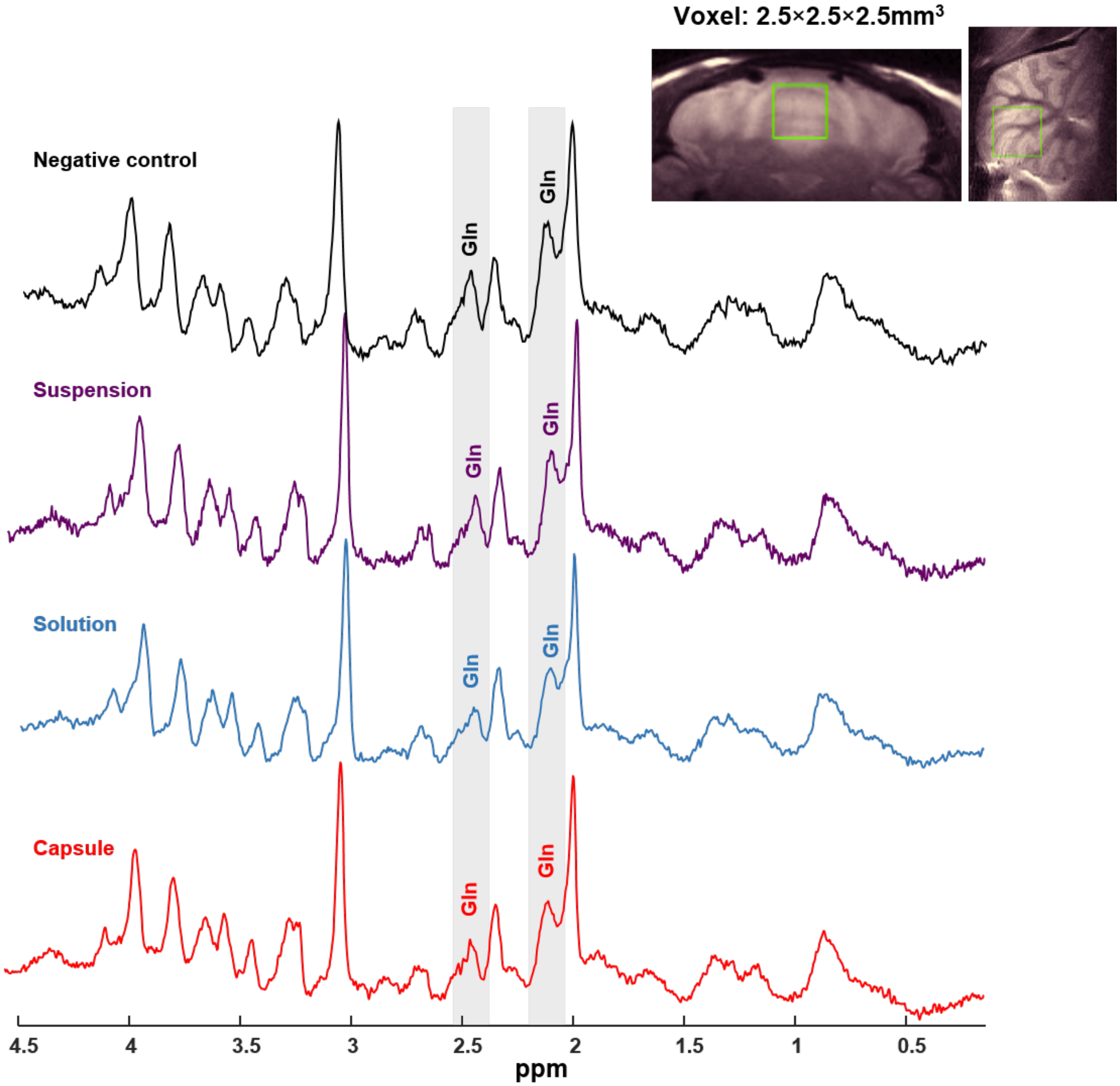

Supplementary Fig. 17. Representative ^{1}H MRS brain spectra measured in the cerebellum of bile duct ligated (BDL) rats. Rats were treated with a drug-free aqueous solution twice daily (negative control), 30 mg/kg 2-octynohydroxamic acid (2-octynoHA) suspension twice daily (suspension), 30 mg/kg 2-octynoHA solution twice daily (solution), 10 mg 2-octynoHA enteric capsule once daily (capsule). The spectra were measured on day 39 post-surgery. The lower glutamine (Gln) in rats treated with 2-octynoHA solution is visible in the spectra. The analyzed brain region is shown as a green rectangle on the T_2-weighted image of the rat's cerebellum.

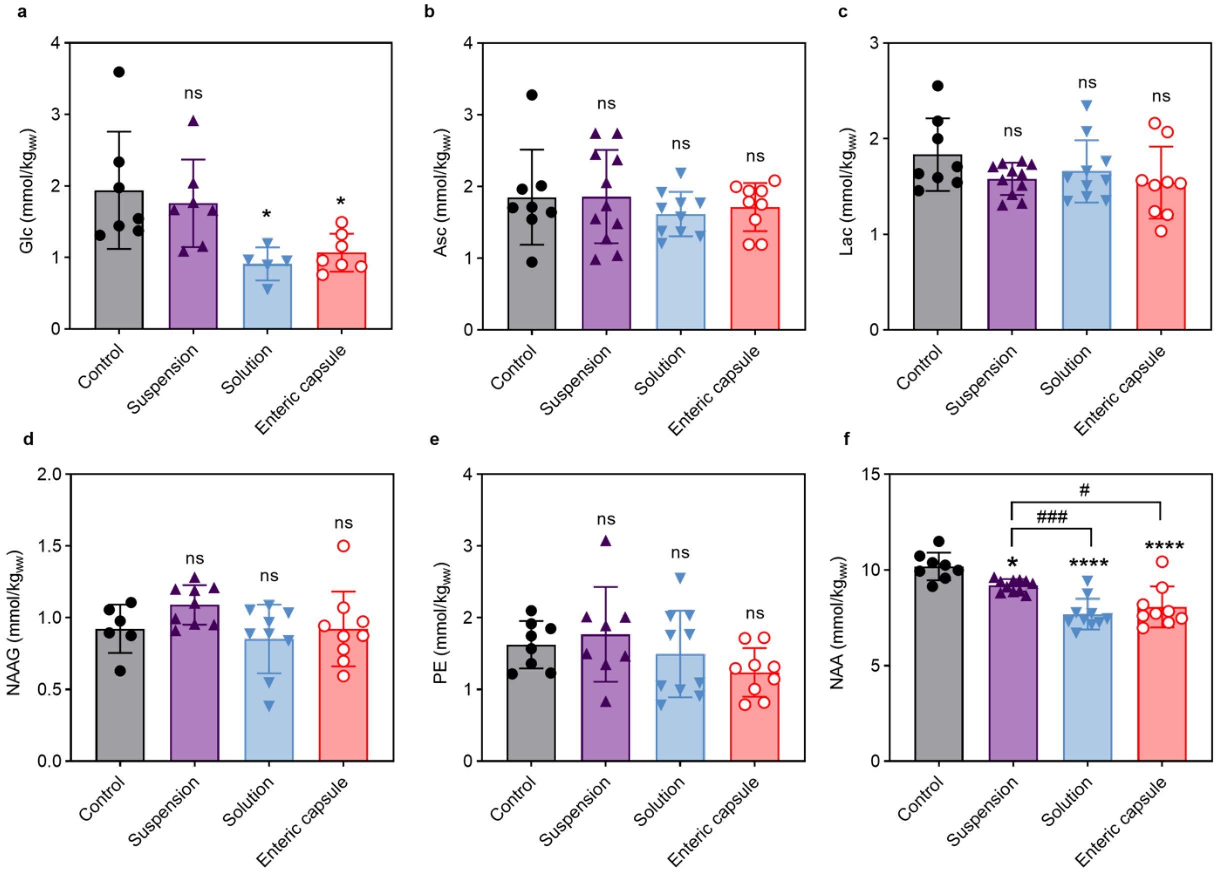

Supplementary Fig. 18. Brain metabolites' levels in bile duct ligated (BDL) rats measured by ^{1}H MRS. Concentrations of **(a)** glucose (Glc), **(b)** ascorbate (Asc), **(c)** lactate (Lac), **(d)** N-acetylaspartylglutamate (NAAG), **(e)** phosphoethanolamine (PE), **(f)** N-acetylaspartate (NAA) after the treatment with the drug-free solution (control), 2-octynoHA suspension (suspension), 2-octynoHA solution (solution), 2-octynoHA enteric capsule (capsule). **(a)** $n = 7$, $n = 7$, $n = 5$, $n = 7$ for the control, suspension, solution and capsule groups, respectively. **(b, c, f)** $n = 8$, $n = 11$, $n = 10$, $n = 9$ for the control, suspension, solution and capsule groups, respectively. **(d)** $n = 6$, $n = 9$, $n = 9$, $n = 9$ for the control, suspension, solution and capsule groups, respectively. **(e)** $n = 8$, $n = 8$, $n = 10$, $n = 9$ for the negative control, suspension, solution and capsule groups, respectively. Data are expressed as mean ± SD from n animals. Statistical significance between groups was calculated by one-way ANOVA with Tukey's multiple comparisons test with $^{*}p < 0.05$, $^{****}p < 0.0001$, ns – not significant vs. control; statistical significance for other comparisons is indicated with $^{\#}p < 0.05$, $^{\#\#\#}p < 0.001$. All p-values are reported in Supplementary Data 20 - 25. Source data are provided as a Source data file.

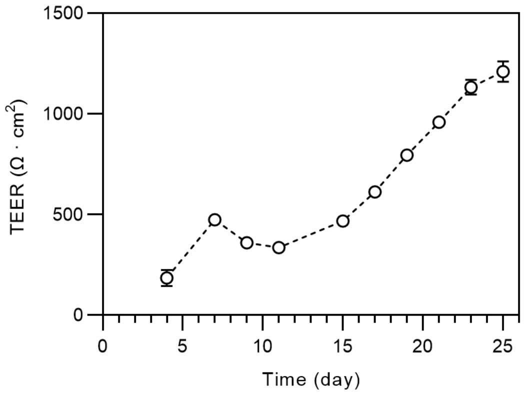

Supplementary Fig. 19. Transepithelial electrical resistance (TEER) of Caco-2 cell monolayers. Evolution of TEER of Caco-2 cell monolayers used for the transport experiment. All monolayers reached at least 1,000 Ω cm^2 TEER starting from day 23 post-seeding (*n* = 6 for days 1 – 23, *n* = 5 for day 25; a single measurement was done for each transwell). Data are expressed as mean ± SD from *n* transwells. Source data are provided as a Source data file.

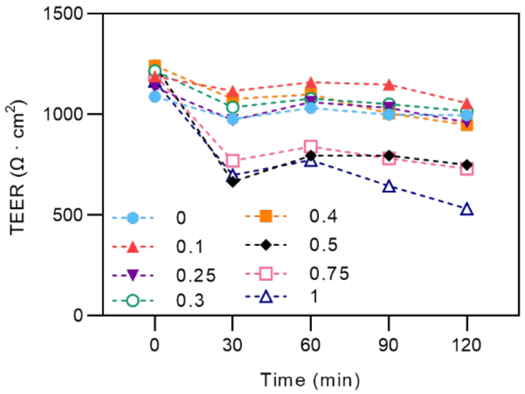

Supplementary Fig. 20. Transepithelial electrical resistance (TEER) of Caco-2 cell monolayers. TEER of Caco-2 monolayers was monitored during 2 h incubation with increasing concentrations of 2-octynoHA (0 – 1 mM). Results of a single measurement are presented. Source data are provided as a Source data file.

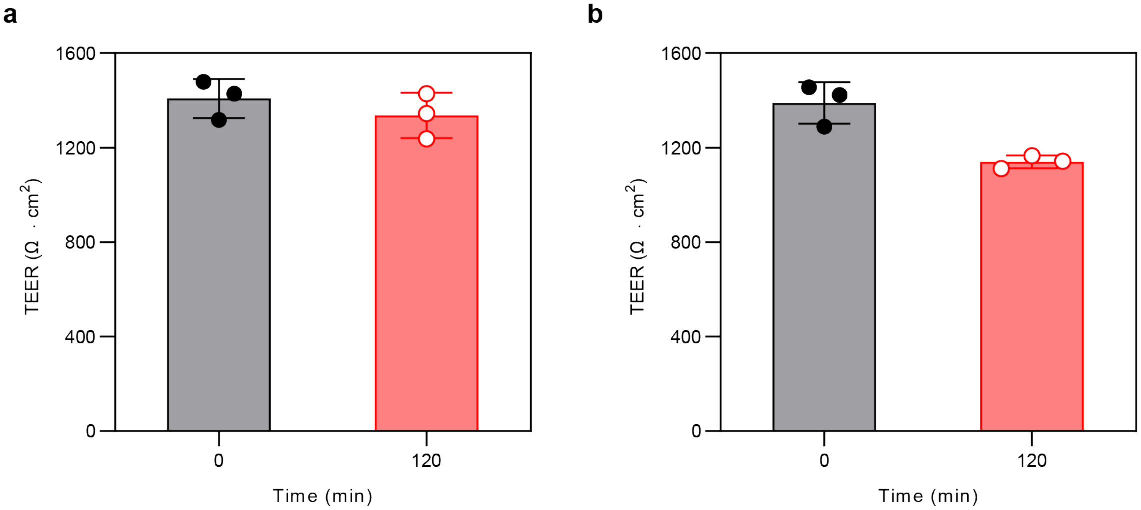

Supplementary Fig. 21. Transepithelial electrical resistance (TEER) of Caco-2 cell monolayers before and after the transport experiment. TEER of monolayers was monitored prior to the treatment and after 2 h incubation with **(a)** 5 µM LY (control) (*n* = 3) or **(b)** a mixture of 0.3 mM 2-octynoHA and 5 µM LY (*n* = 3). TEER after incubation with LY was 1337 ± 96 Ω cm^2 vs. 1409 ± 82 Ω cm^2 before the treatment. After incubation with a mixture of 2-octynoHA and LY, TEER decreased from 1390 ± 88 Ω cm^2 to 1140 ± 27 Ω cm^2. Data are expressed as mean ± SD from *n* transwells. Source data are provided as a Source data file.

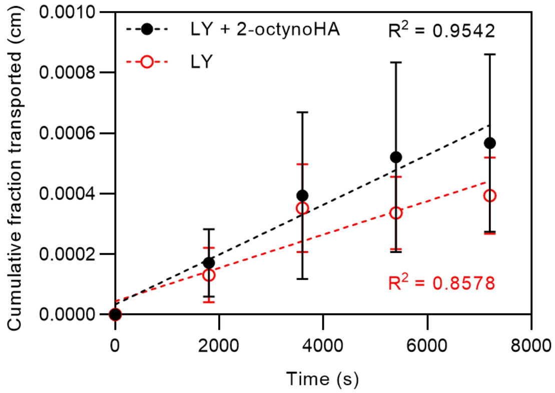

Supplementary Fig. 22. Permeability of Lucifer Yellow (LY) across Caco-2 monolayers. Cumulative fraction transported for LY (paracellular marker) co-incubated with 0.3 mM 2-octynoHA (black line) or without the inhibitor (red line). The apparent permeability coefficient (P_{app}) was calculated from the slope of the linear fit indicated as a dashed line. P_{app} of LY co-incubated with 2-octynoHA was 8.2 ± 4.5 x 10^{-8} cm s^{-1} (*n*=3) vs. 5.5 ± 1.6 x 10^{-8} cm s^{-1} (*n*=3) without the inhibitor. Data are expressed as mean ± SD from *n* monolayers. Source data are provided as a Source data file.

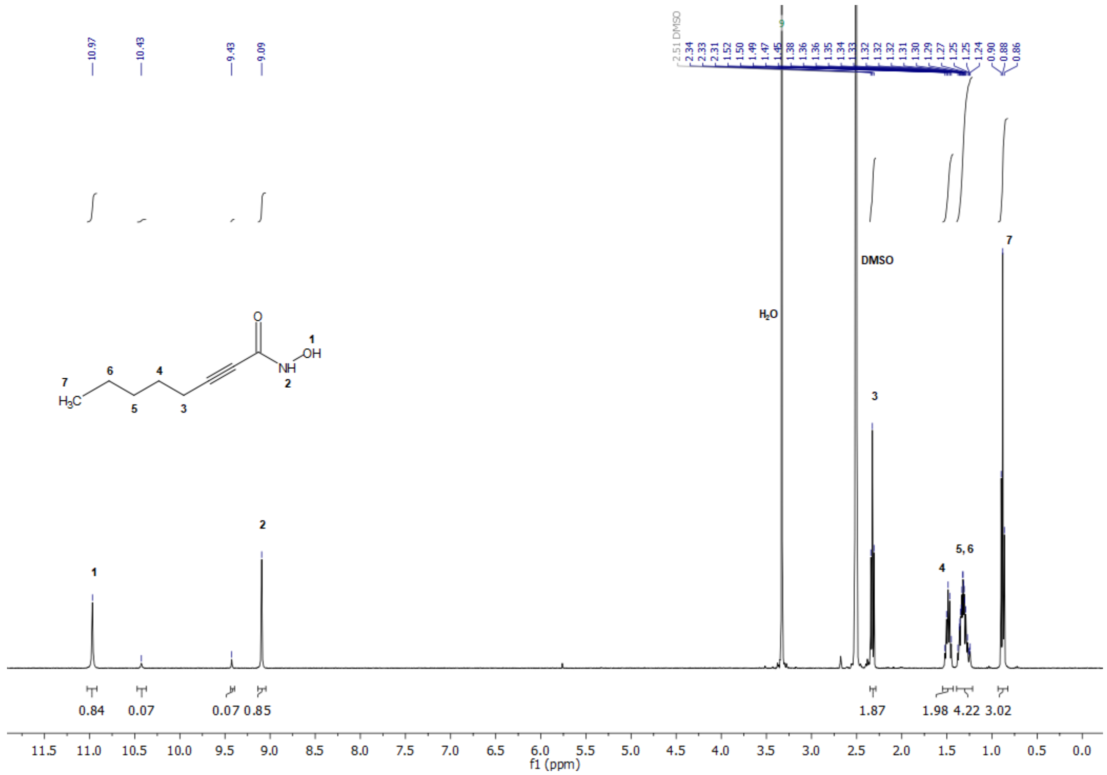

Supplementary Fig. 23. ^{1}H NMR spectrum of 2-octynoHA.

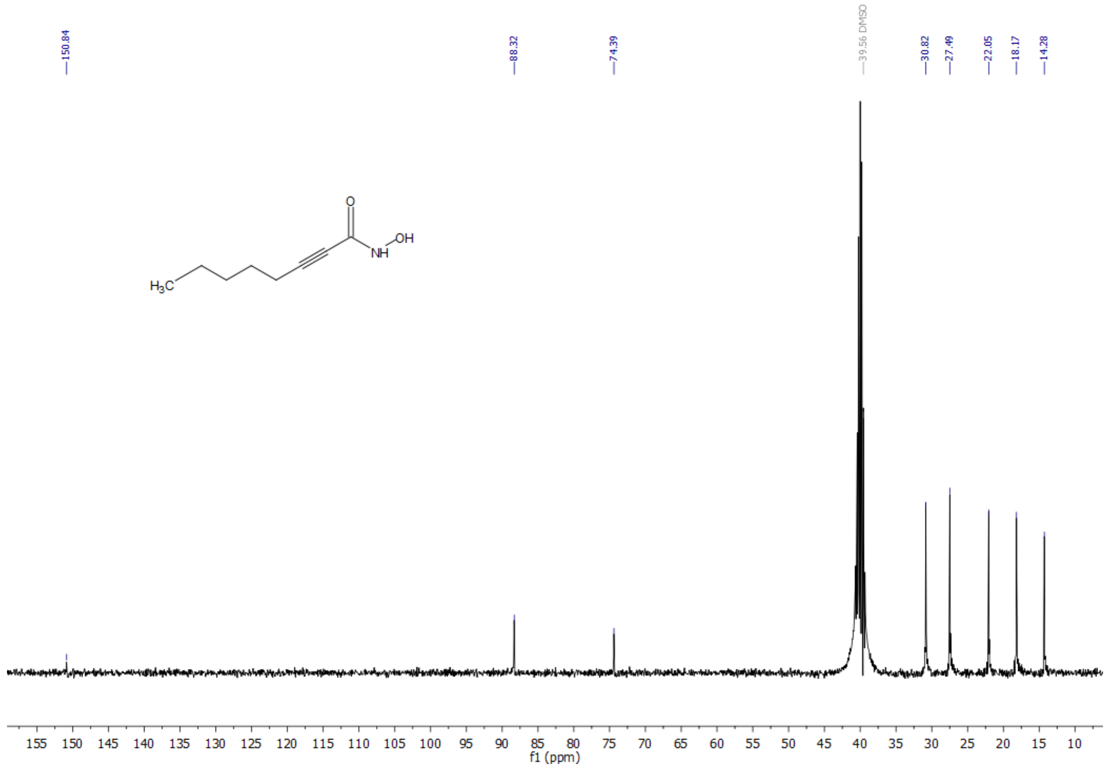

Supplementary Fig. 24. ^{13}C NMR spectrum of 2-octynoHA.

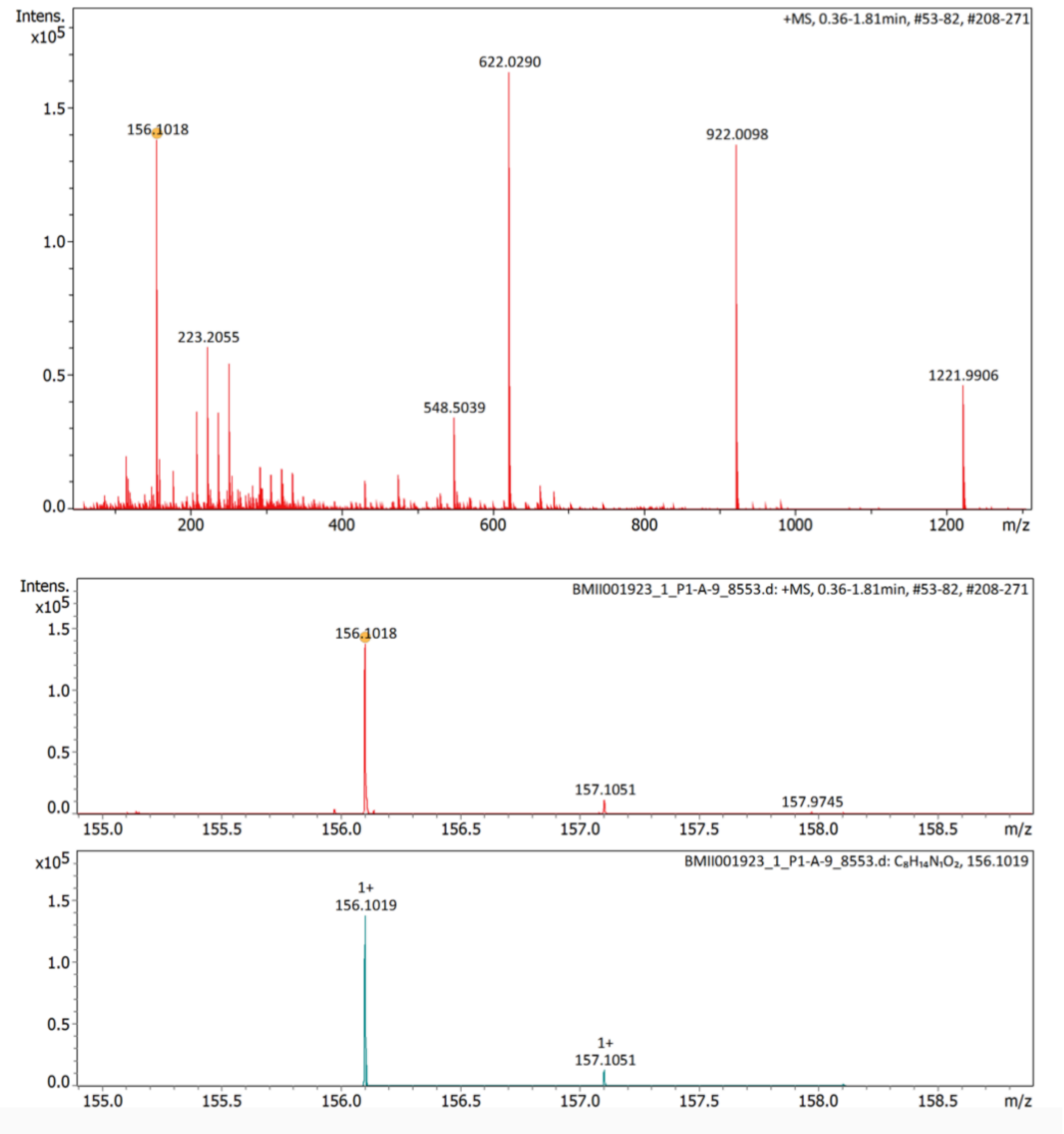

Supplementary Fig. 25. HRMS spectrum of 2-octynoHA.

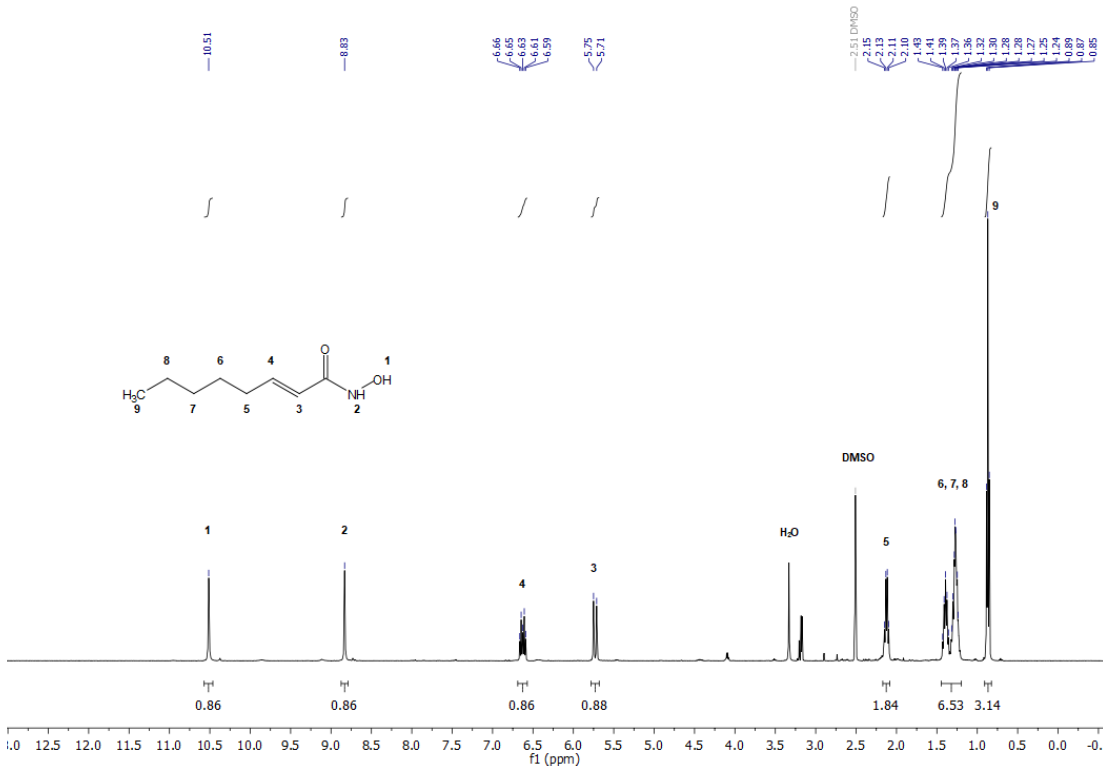

Supplementary Fig. 26. ^{1}H NMR spectrum of 2-octenoHA.

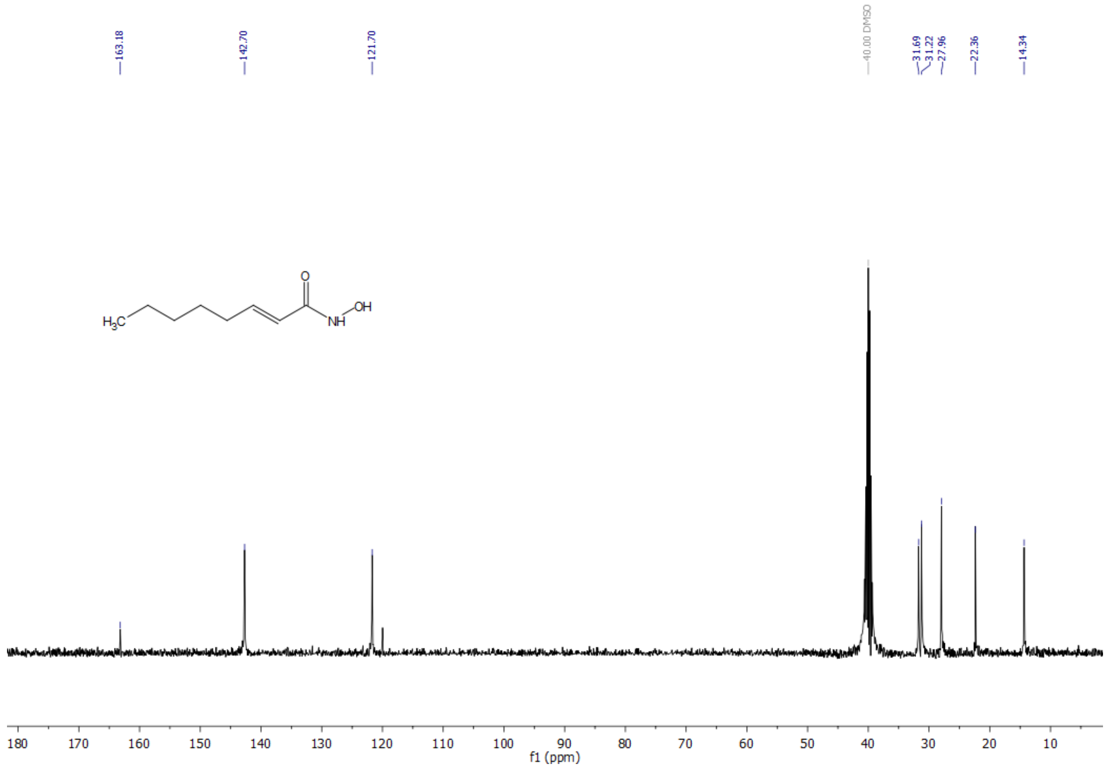

Supplementary Fig. 27. ^{13}C NMR spectrum of 2-octenoHA.

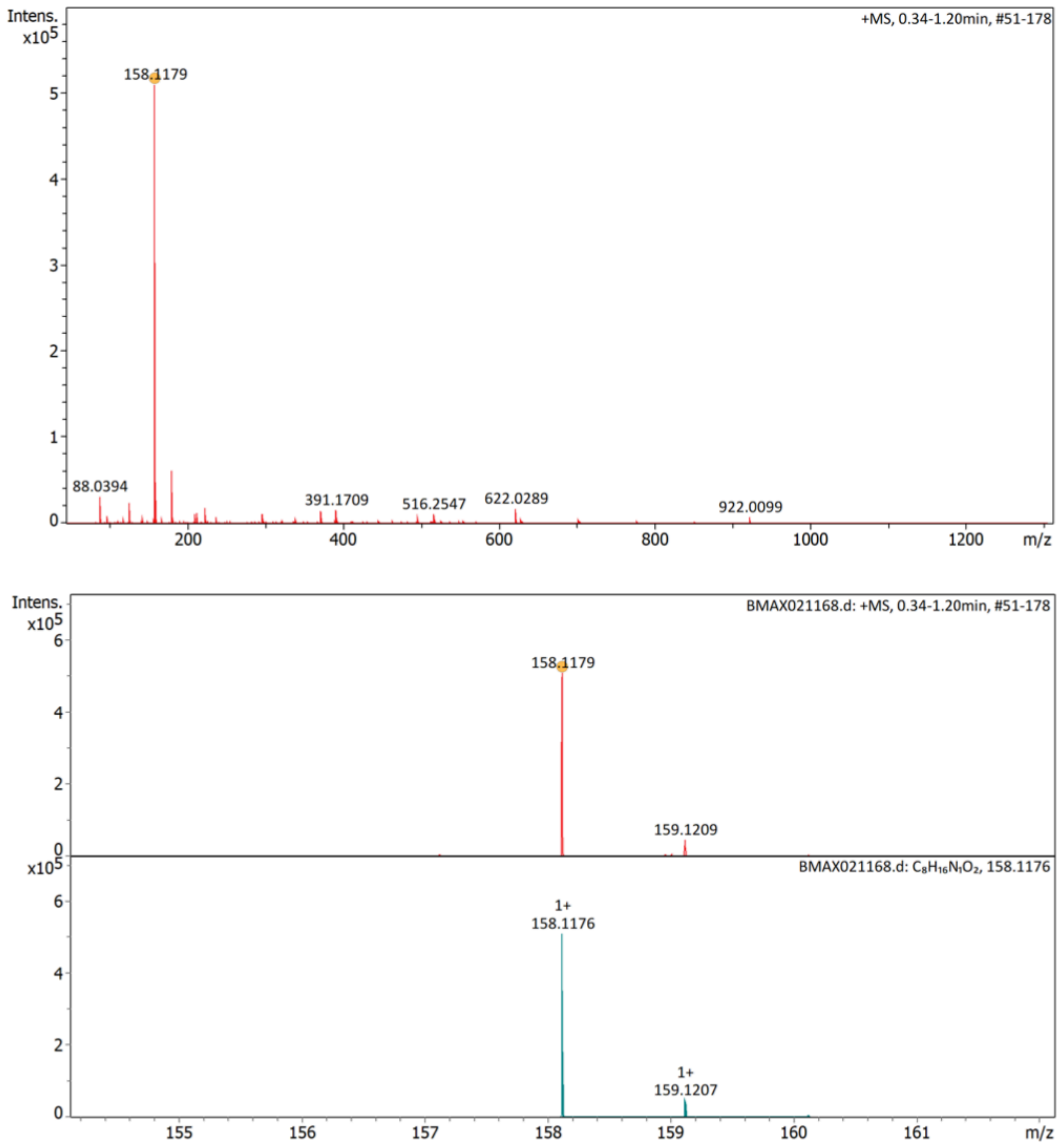

Supplementary Fig. 28. HRMS spectrum of 2-octenoHA.

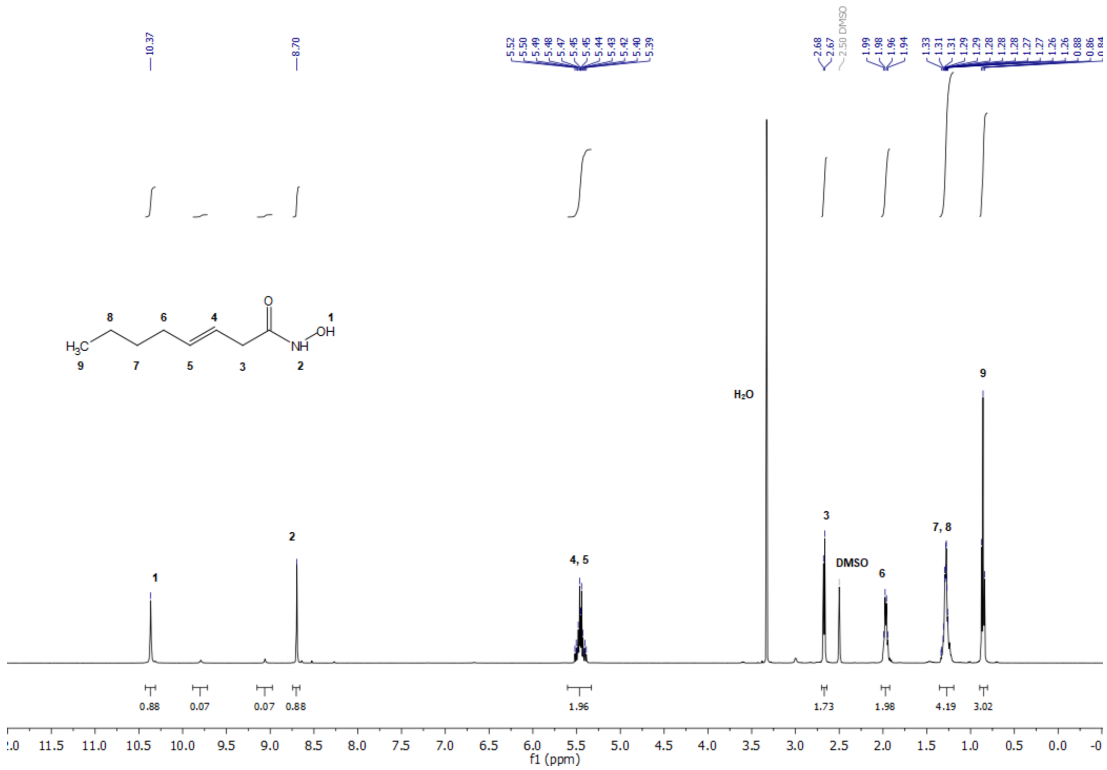

Supplementary Fig. 29. ^{1}H NMR spectrum of 3-octenoHA.

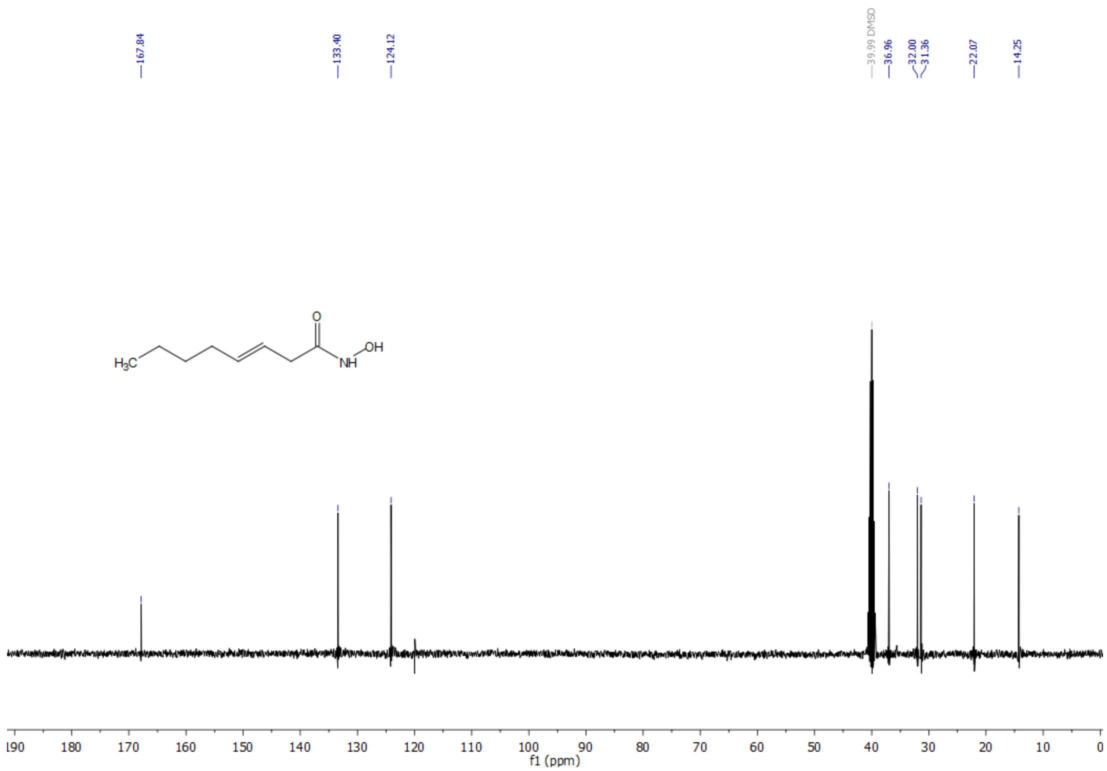

Supplementary Fig. 30. ^{13}C NMR spectrum of 3-octenoHA.

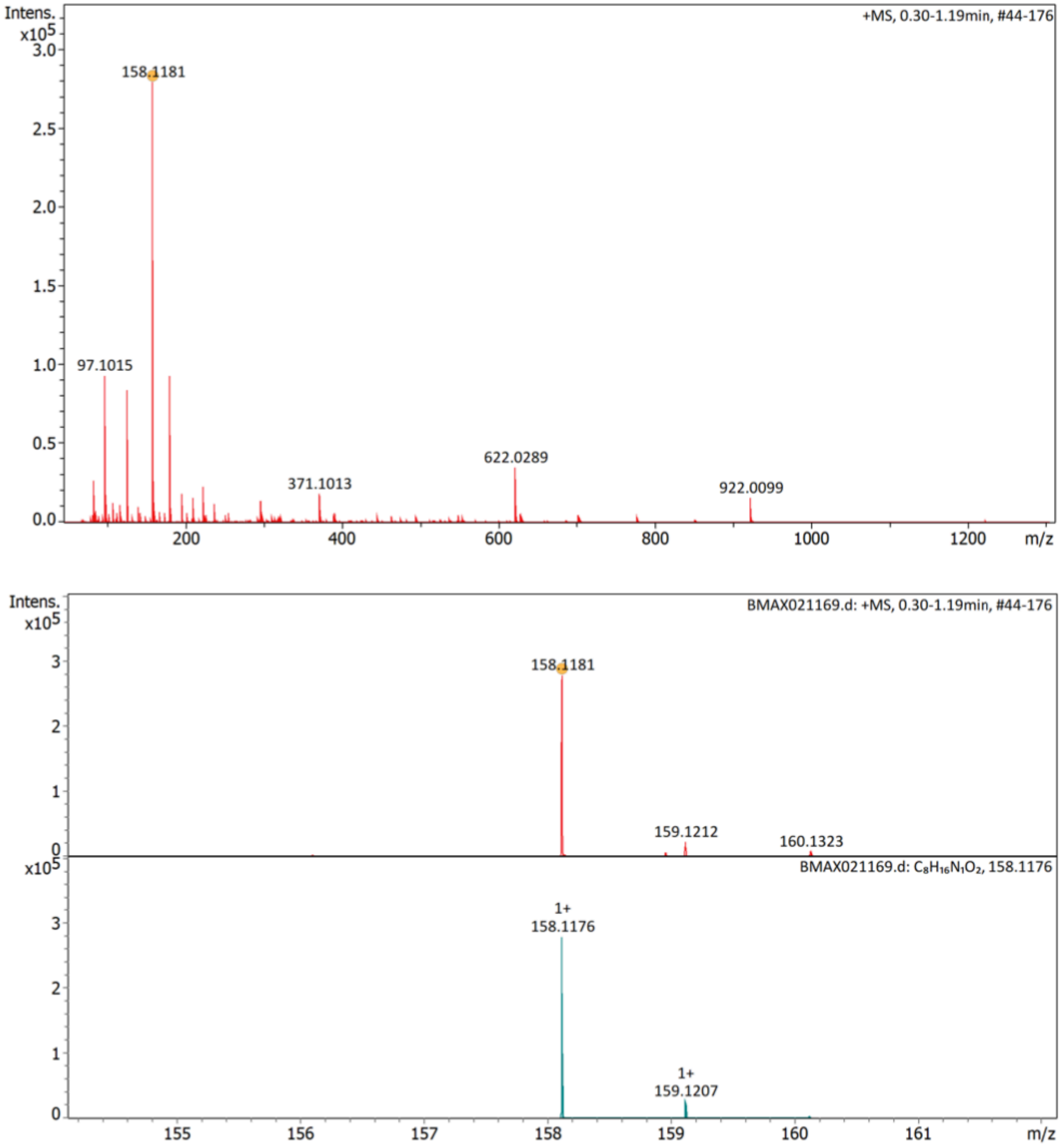

Supplementary Fig. 31. HRMS spectrum of 3-octenoHA.

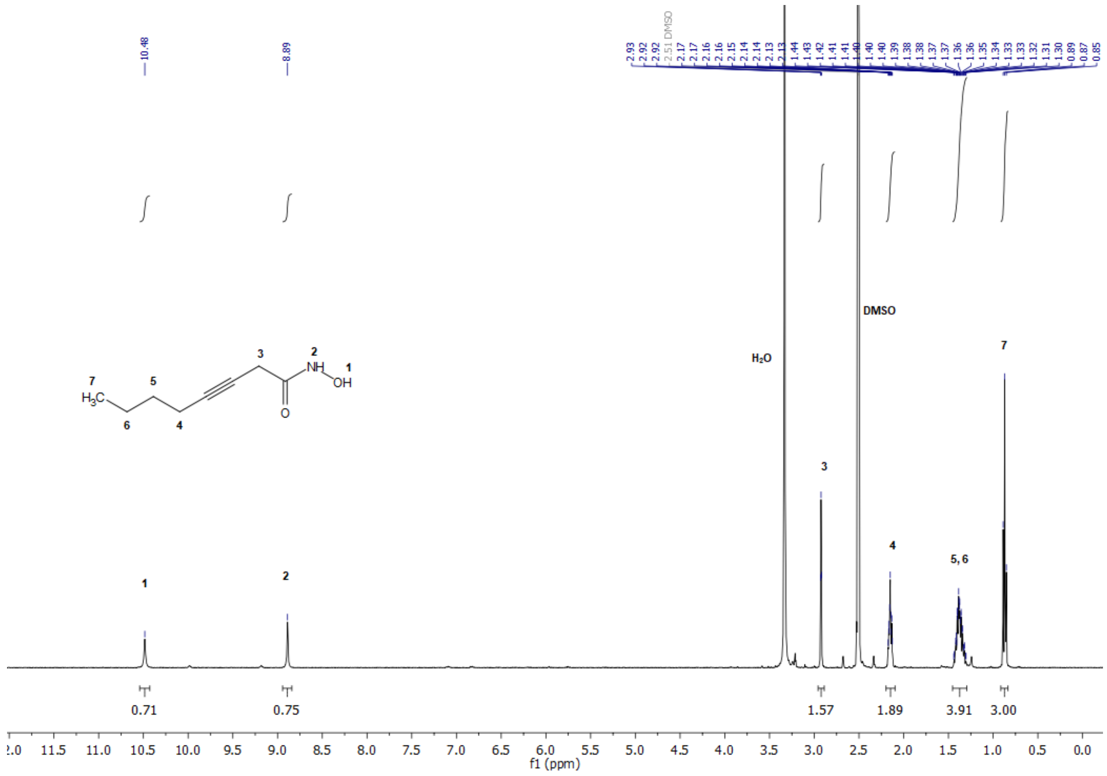

Supplementary Fig. 32. ^{1}H NMR spectrum of 3-octynoHA.

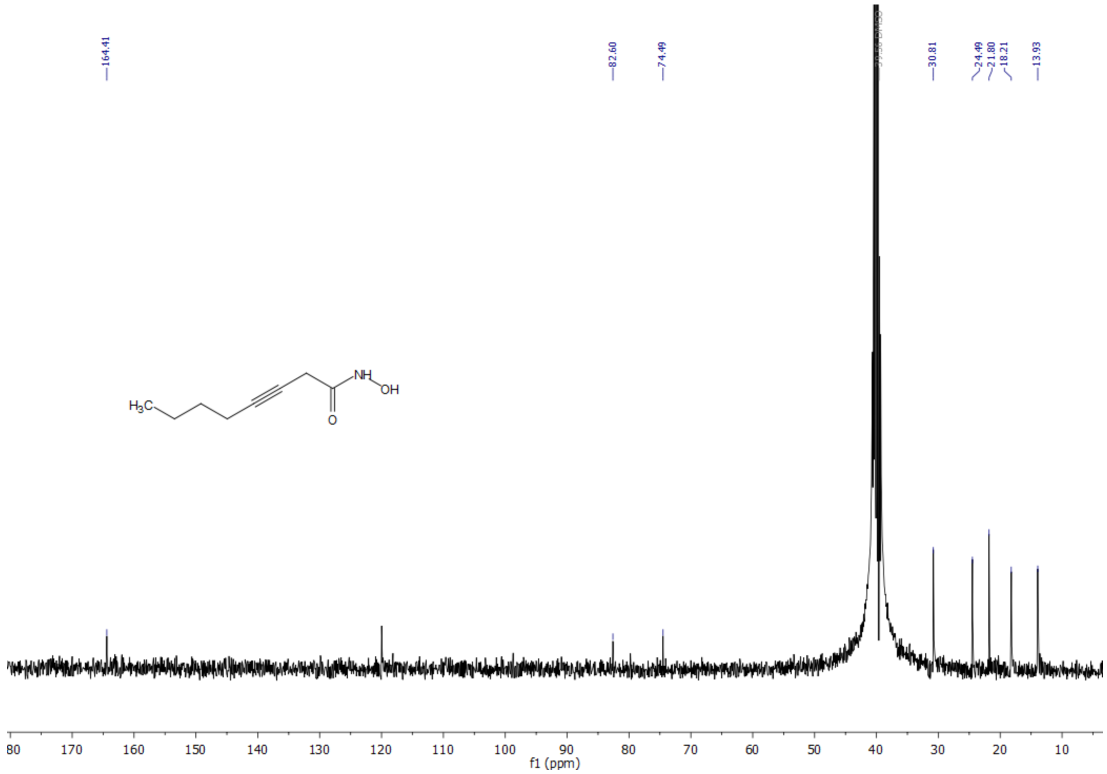

Supplementary Fig. 33. ^{13}C NMR spectrum of 3-octynoHA.

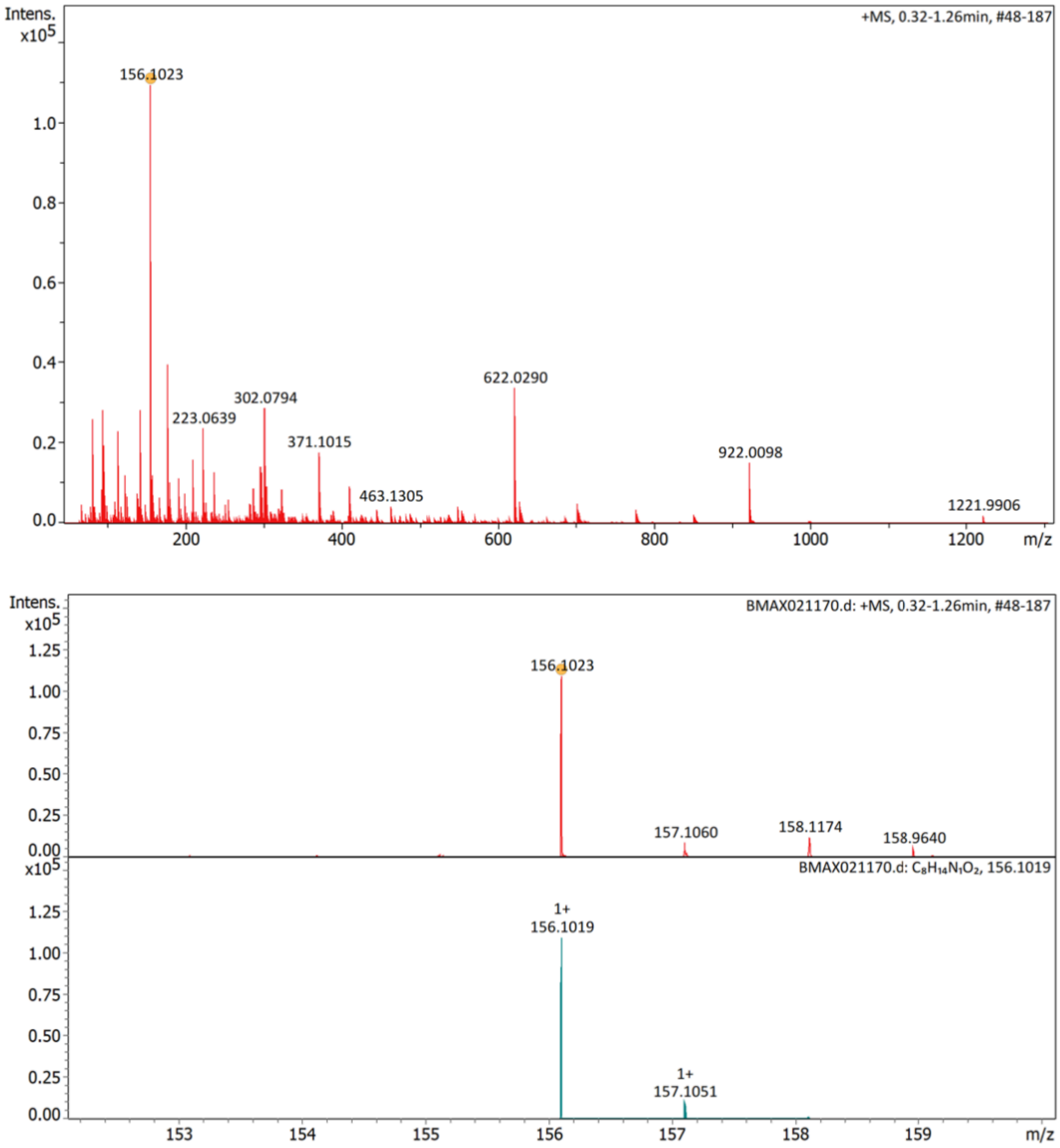

Supplementary Fig. 34. HRMS spectrum of 3-octynoHA.

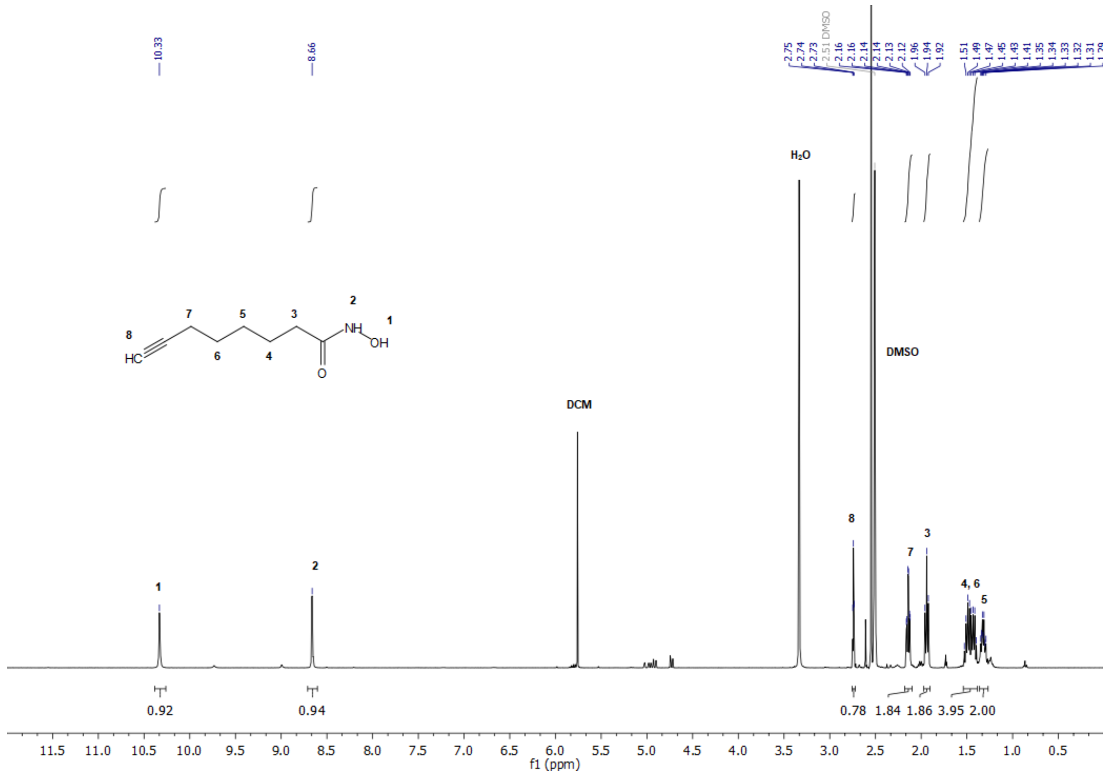

Supplementary Fig. 35. ^{1}H NMR spectrum of 7-octynoHA.

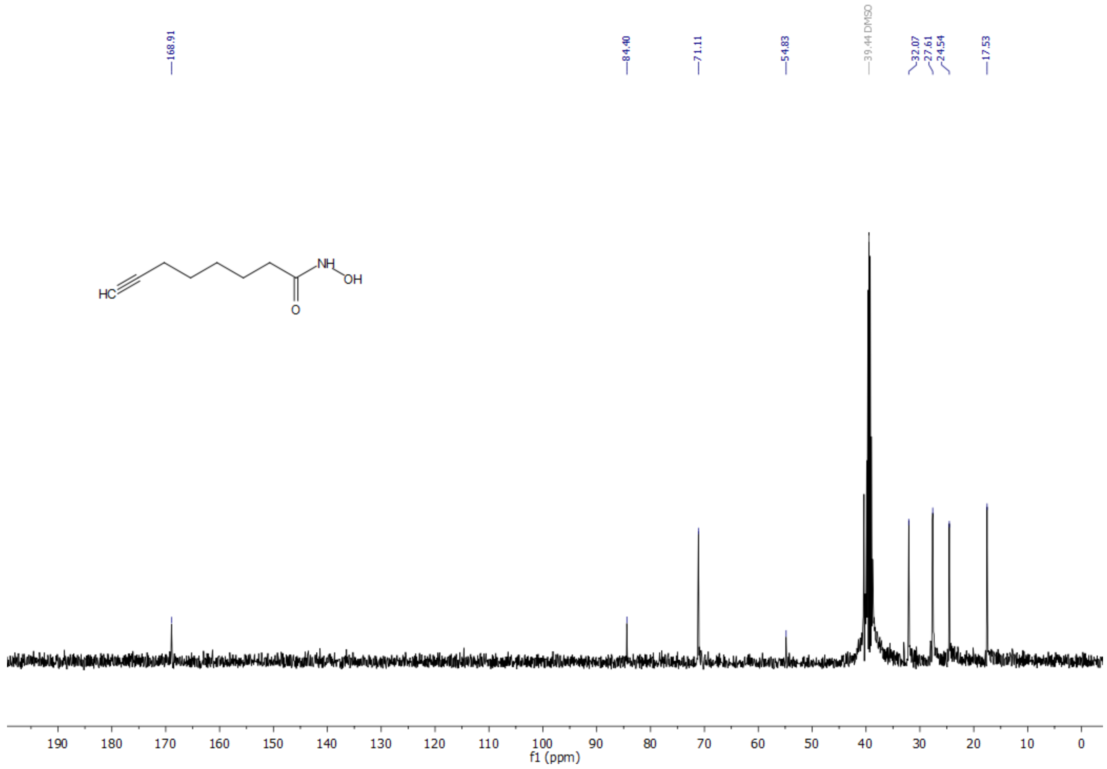

Supplementary Fig. 36. ^{13}C NMR spectrum of 7-octynoHA.

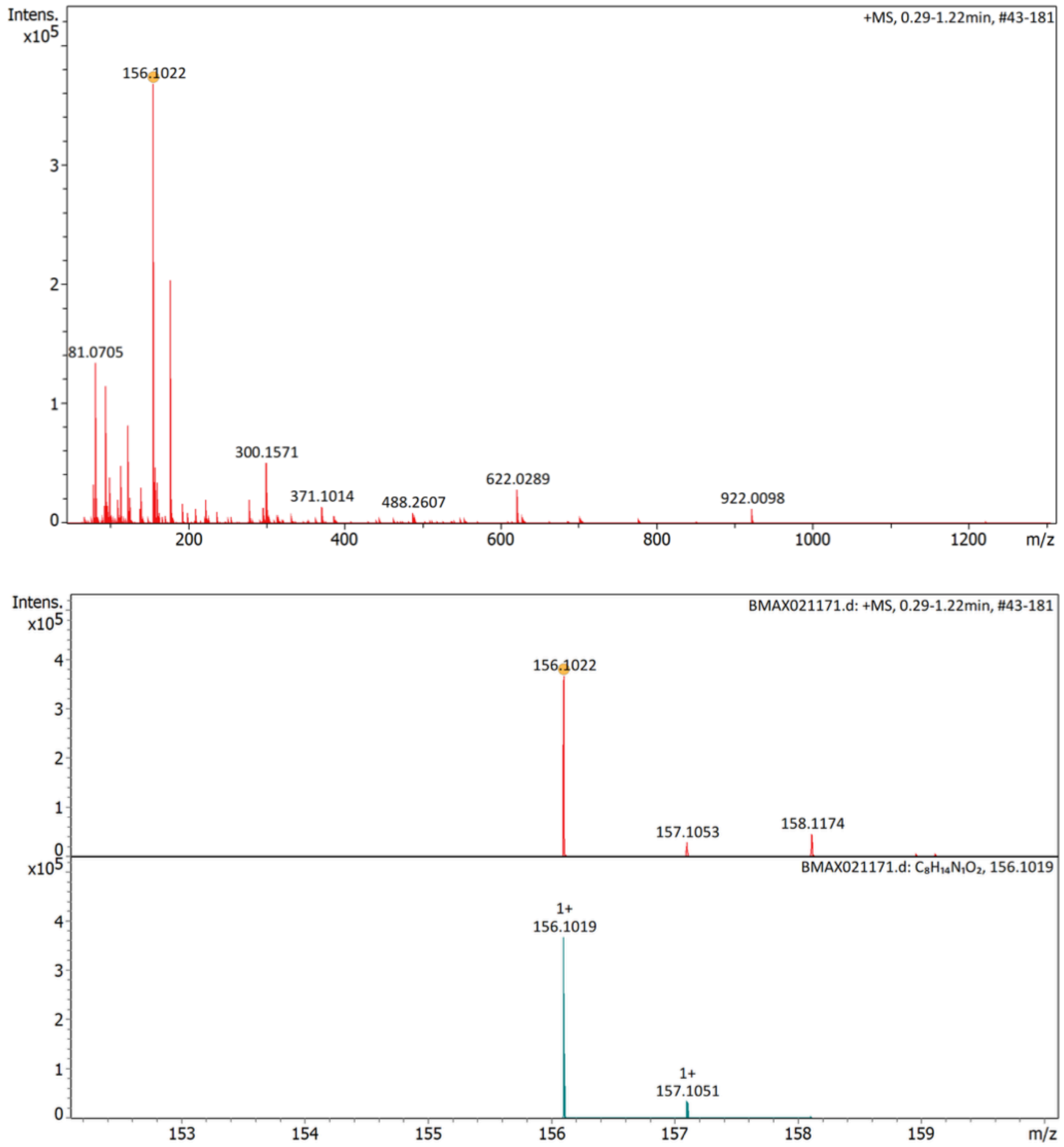

Supplementary Fig. 37. HRMS spectrum of 7-octynoHA.

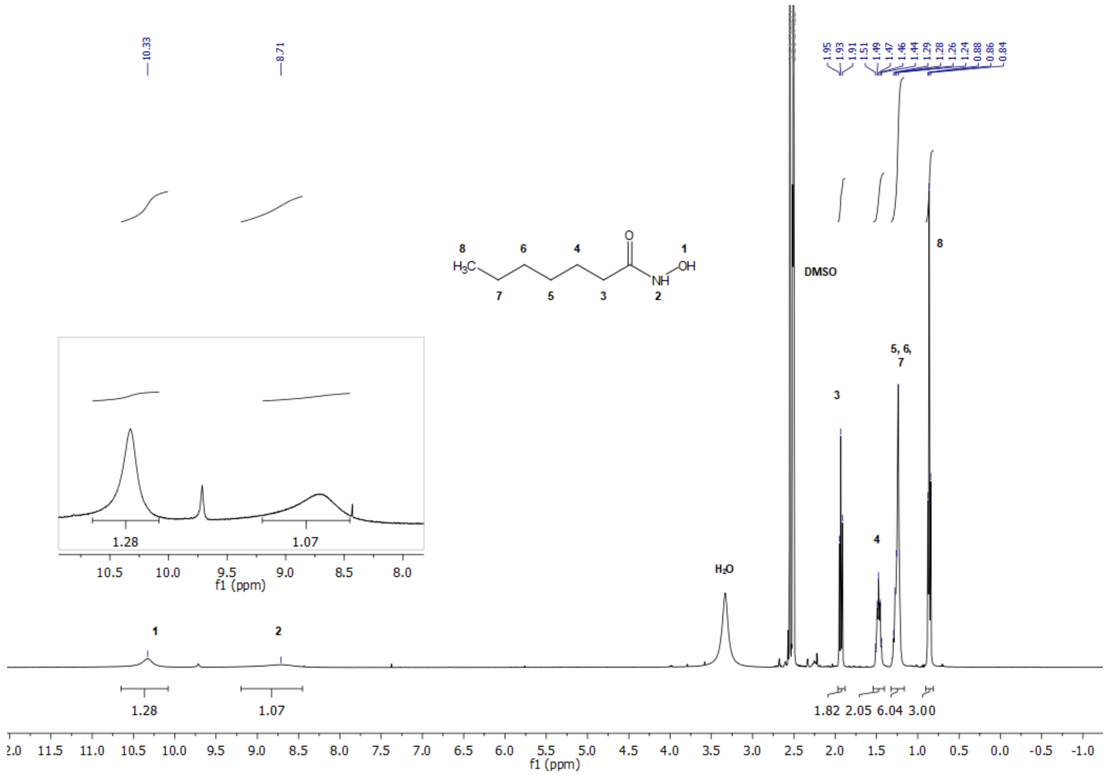

Supplementary Fig. 38. ^{1}H NMR spectrum of HHA.

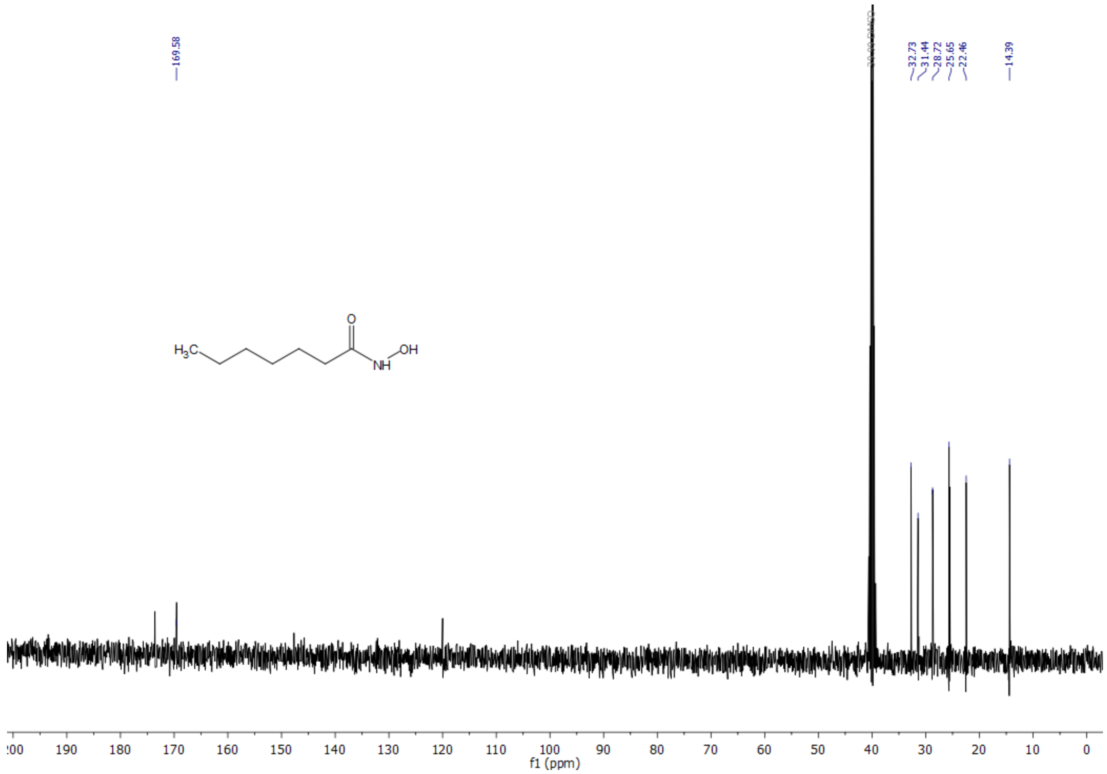

Supplementary Fig. 39. ^{13}C NMR spectrum of HHA.

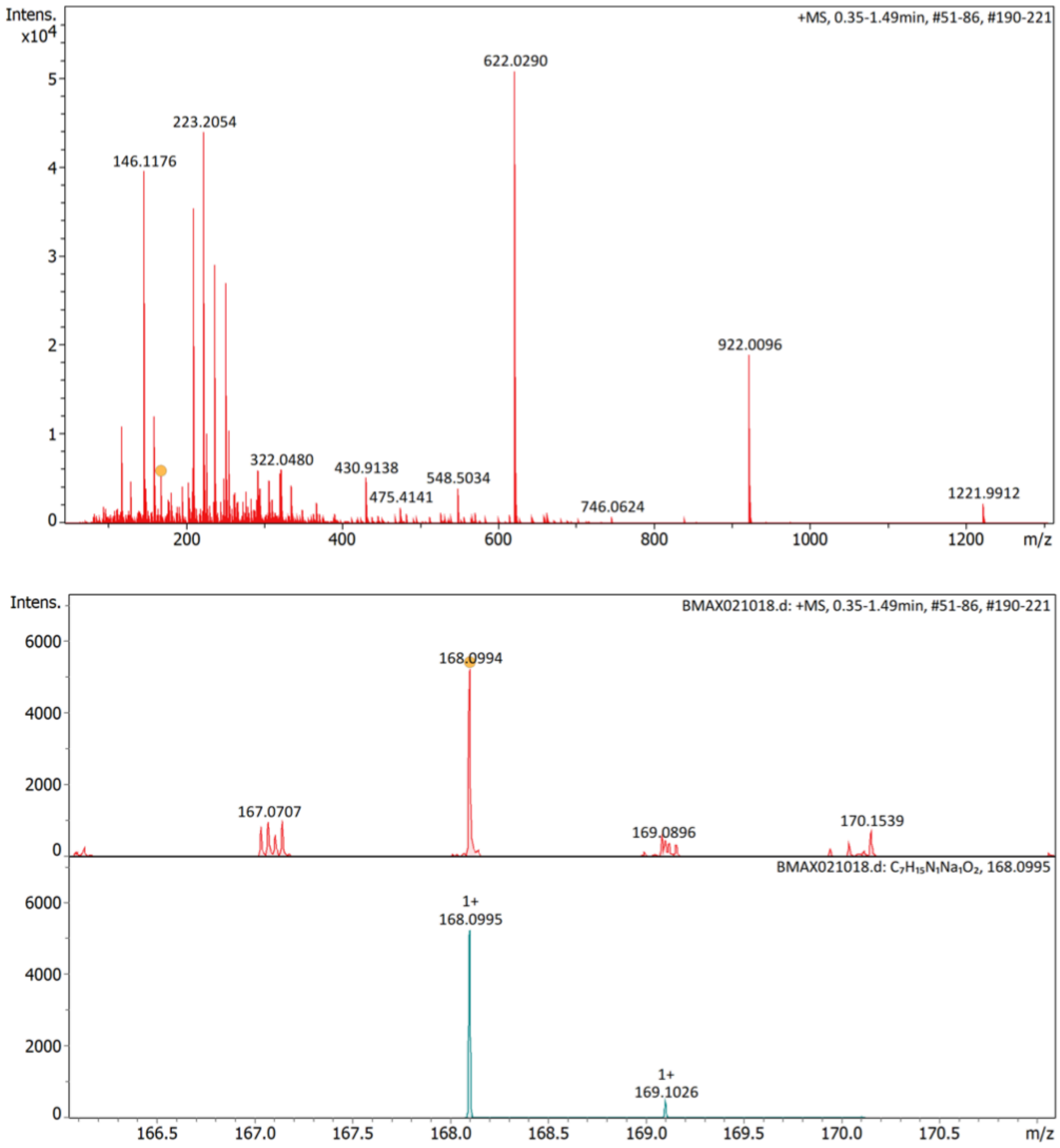

Supplementary Fig. 40. HRMS spectrum of HHA.

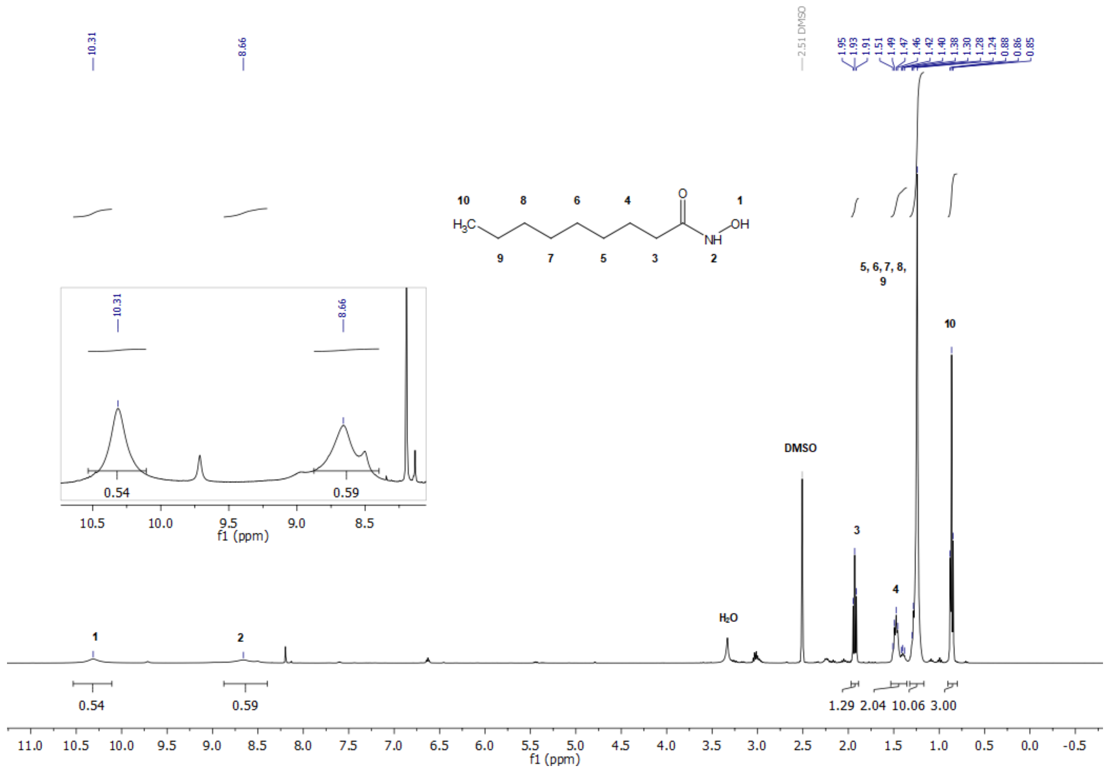

Supplementary Fig. 41. ^{1}H NMR spectrum of NHA.

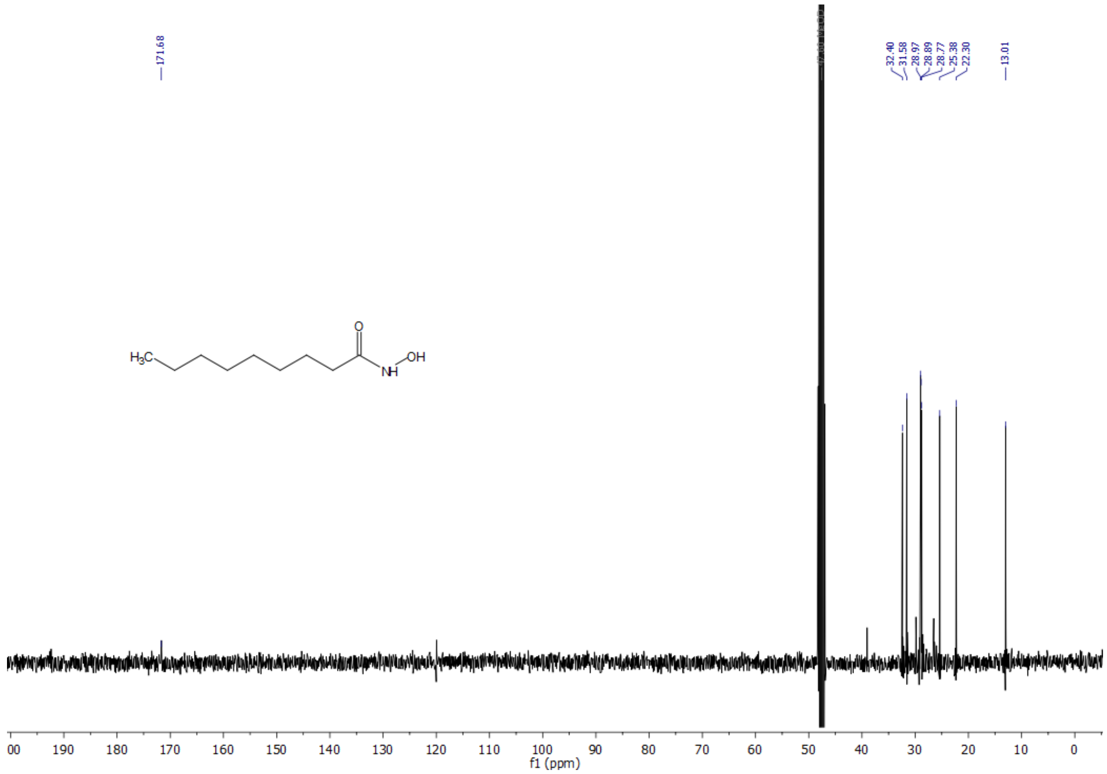

Supplementary Fig. 42. ^{13}C NMR spectrum of NHA.

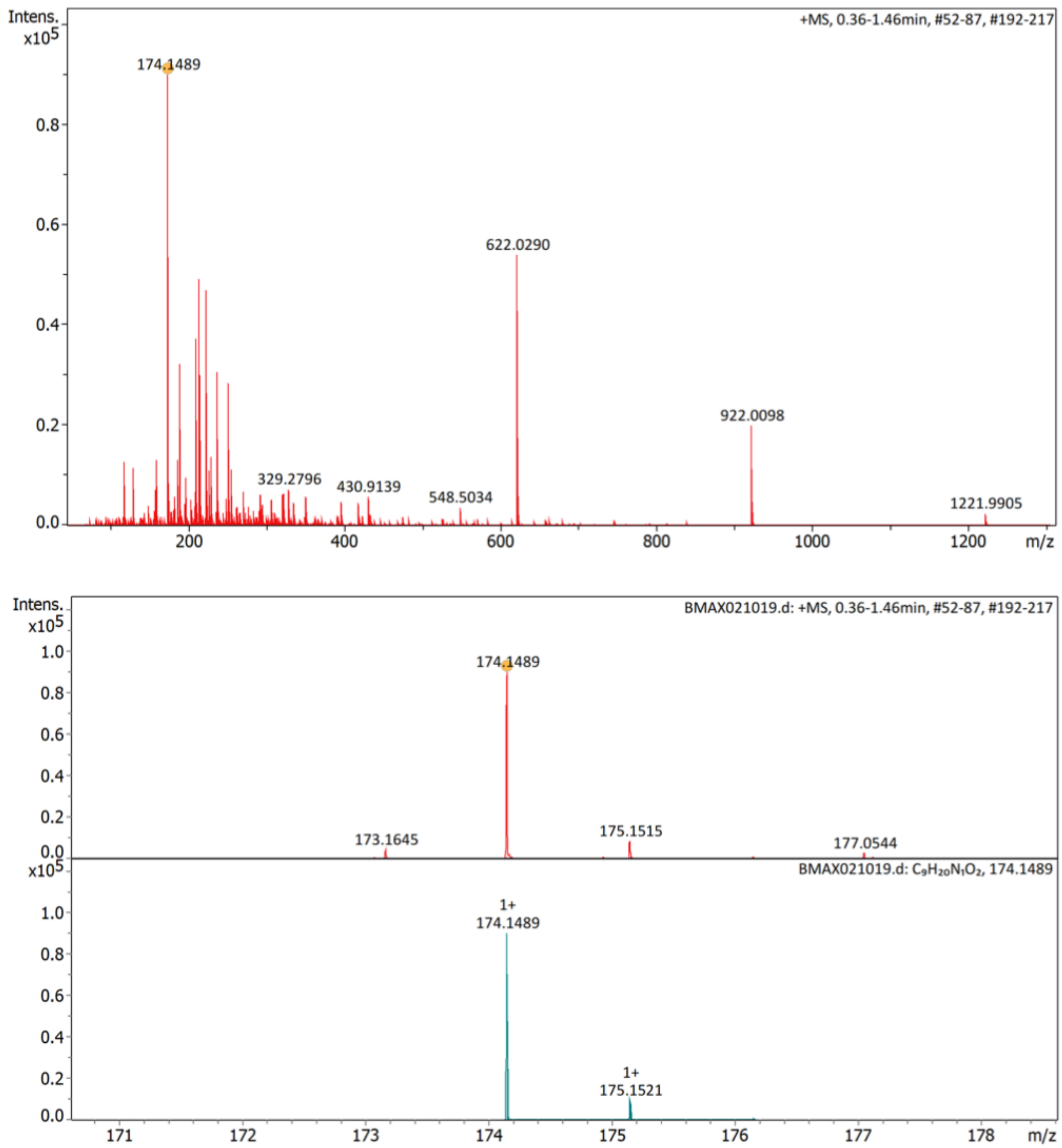

Supplementary Fig. 43. HRMS spectrum of NHA.

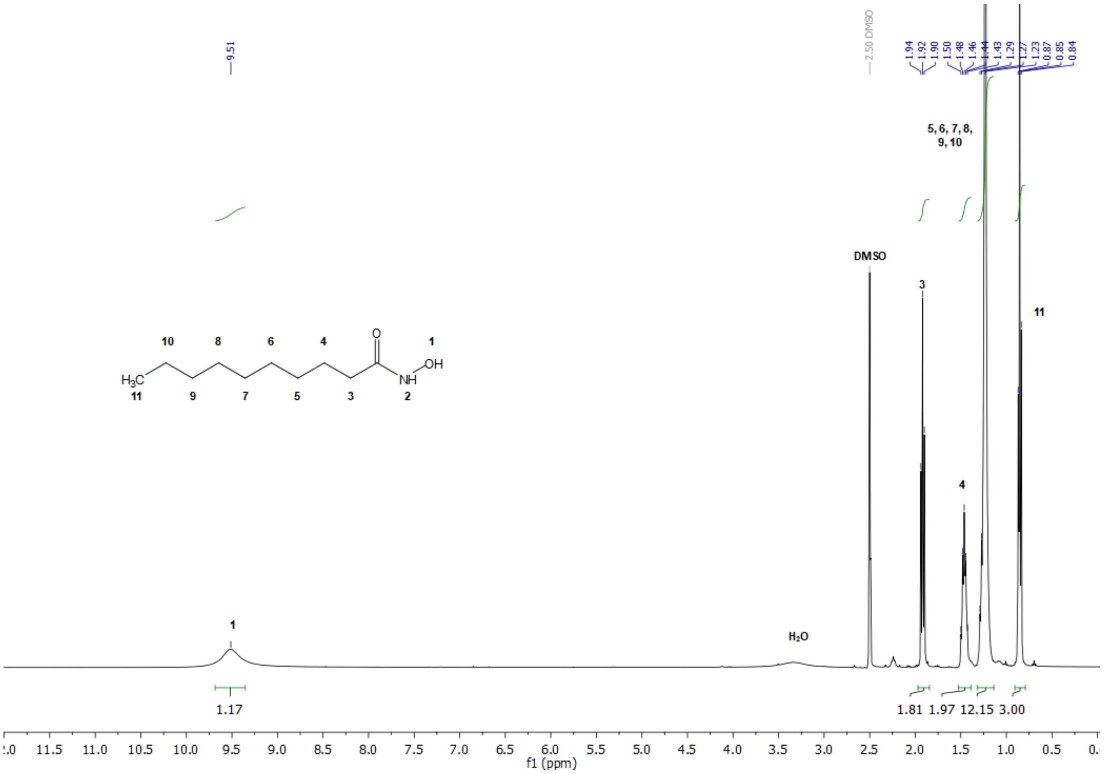

Supplementary Fig. 44. ^{1}H NMR spectrum of DHA.

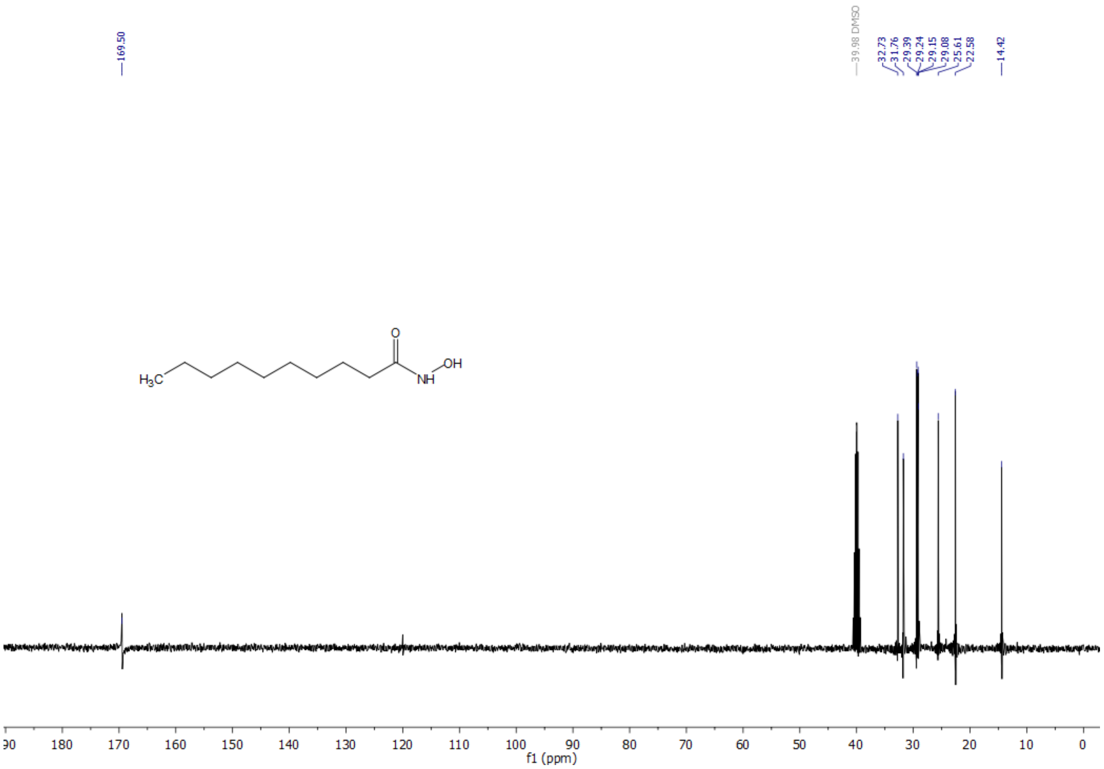

Supplementary Fig. 45. ^{13}C NMR spectrum of DHA.

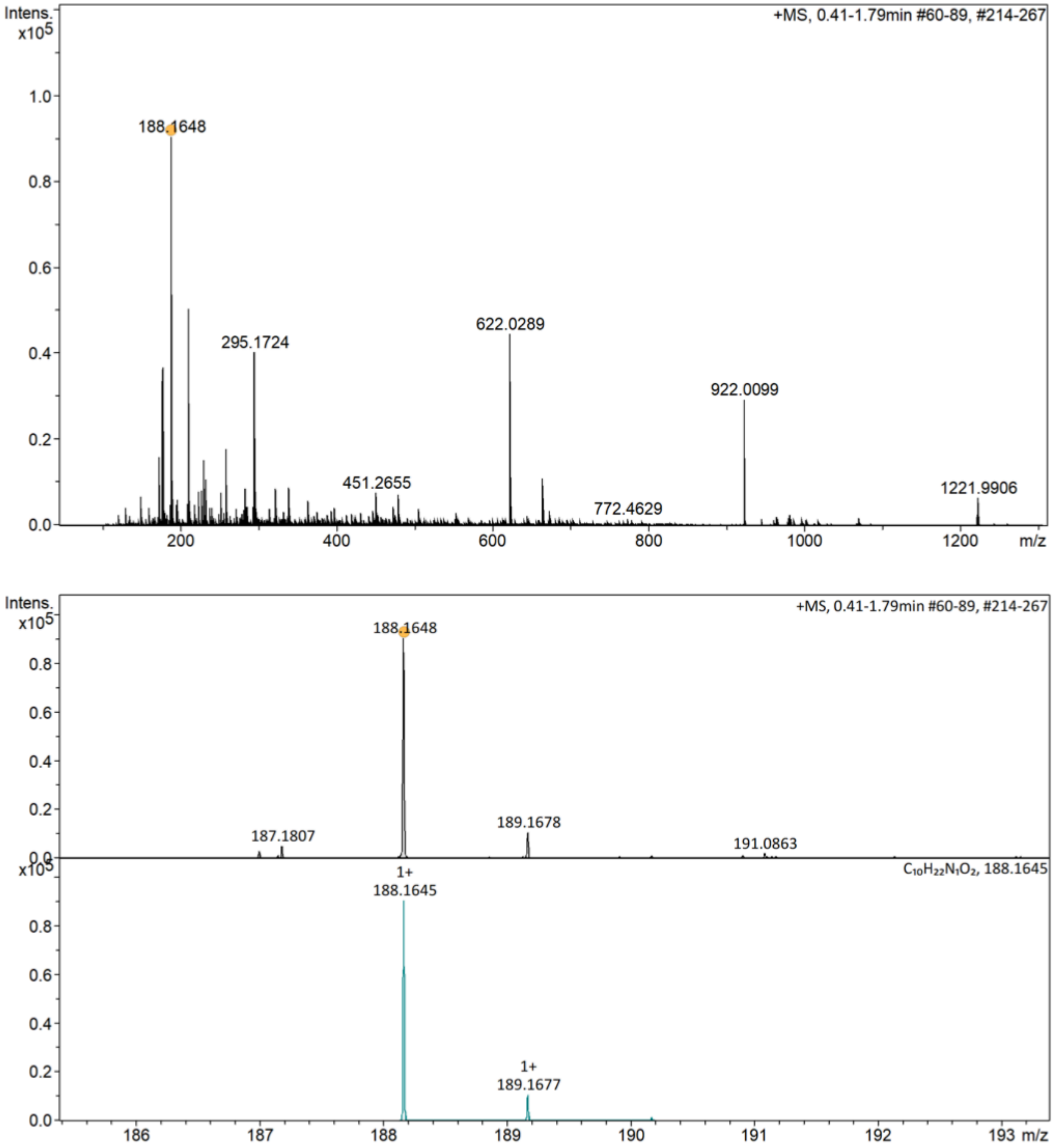

Supplementary Fig. 46. HRMS spectrum of DHA.

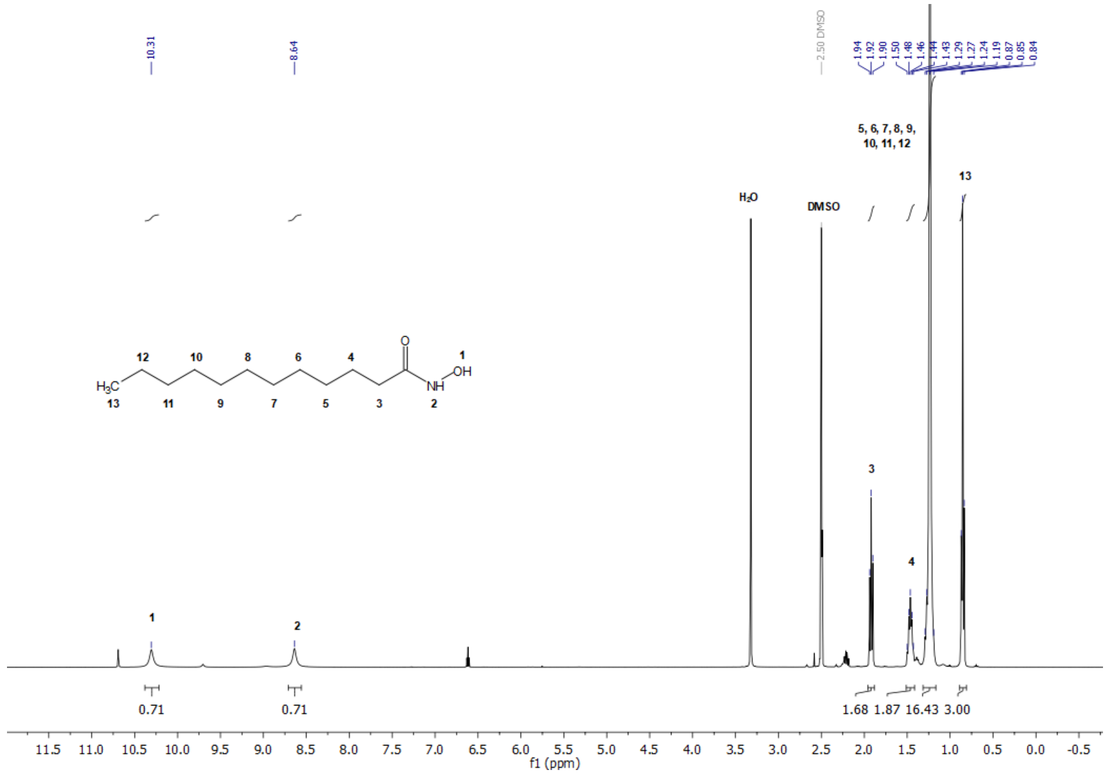

Supplementary Fig. 47. ^{1}H NMR spectrum of LHA.

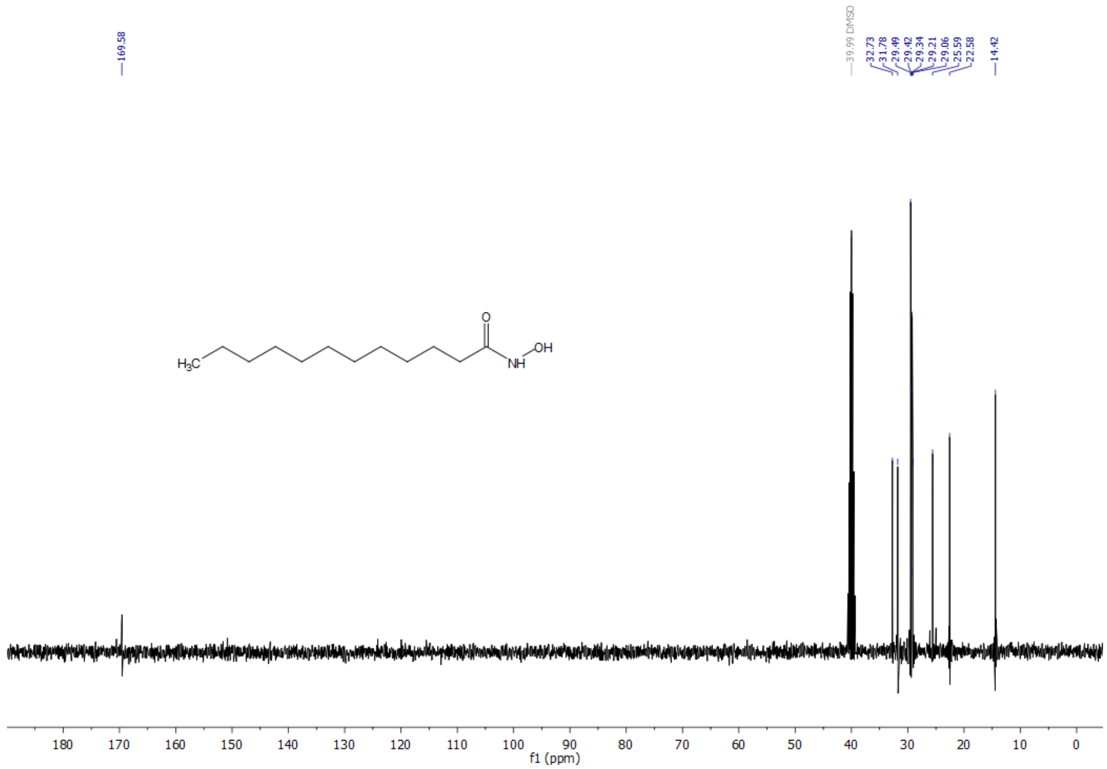

Supplementary Fig. 48. ^{13}C NMR spectrum of LHA.

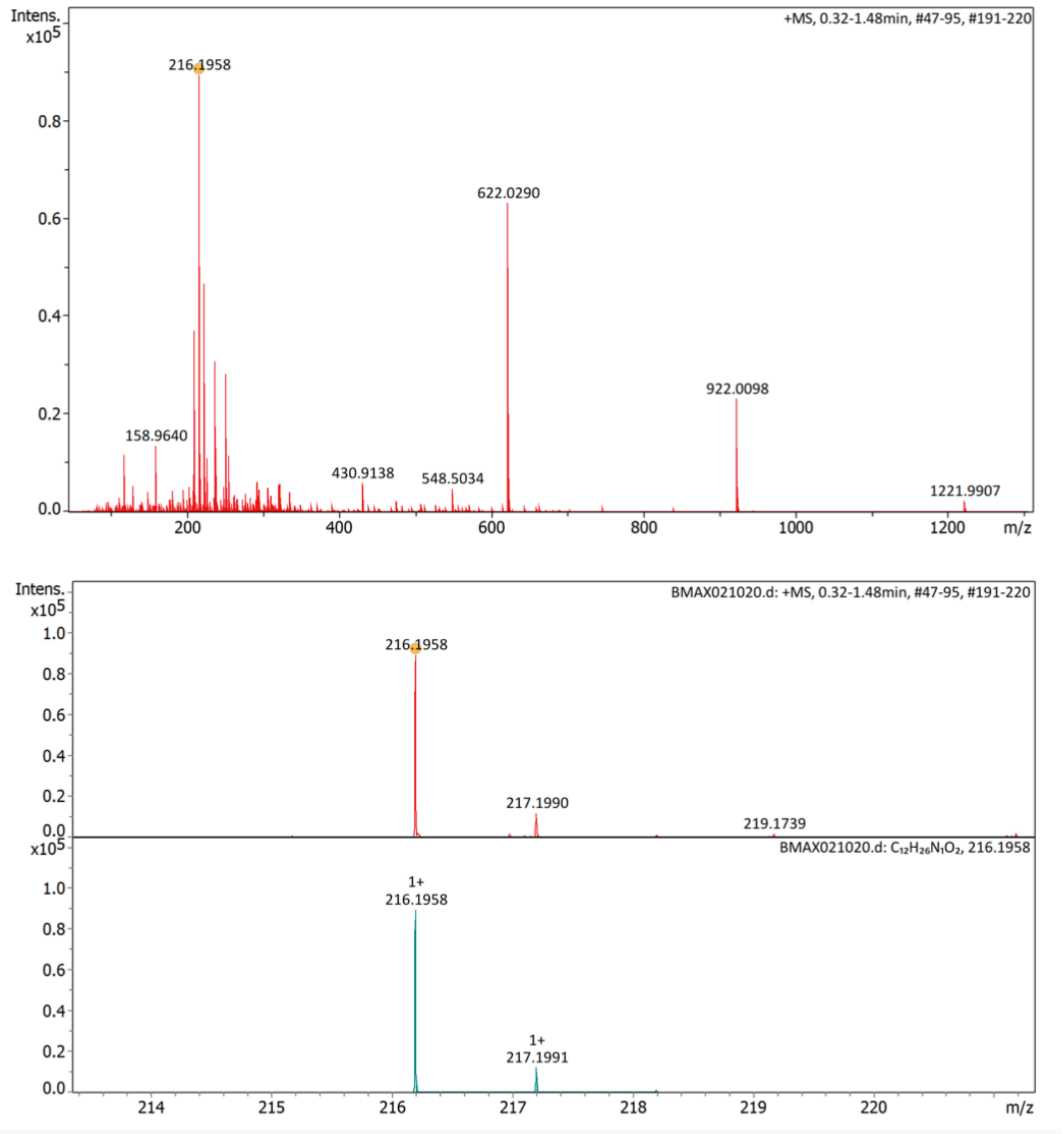

Supplementary Fig. 49. HRMS spectrum of LHA.

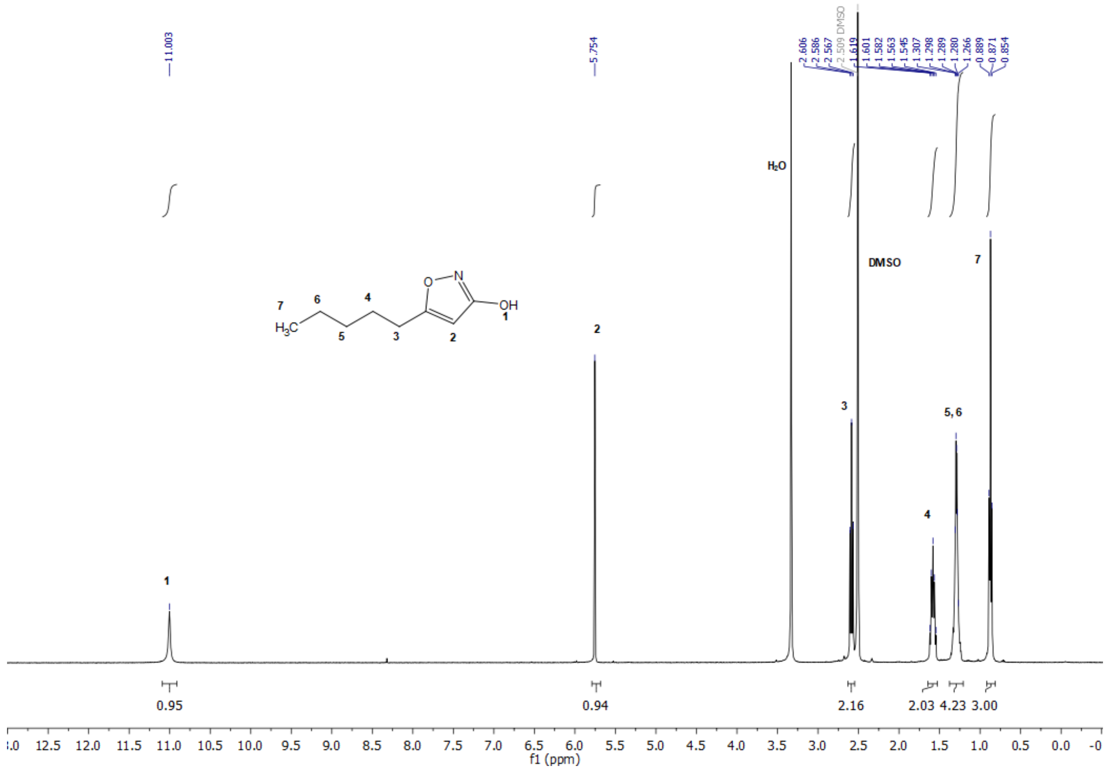

Supplementary Fig. 50. ^{1}H NMR spectrum of 5-pentylisoxazol-3-ol.

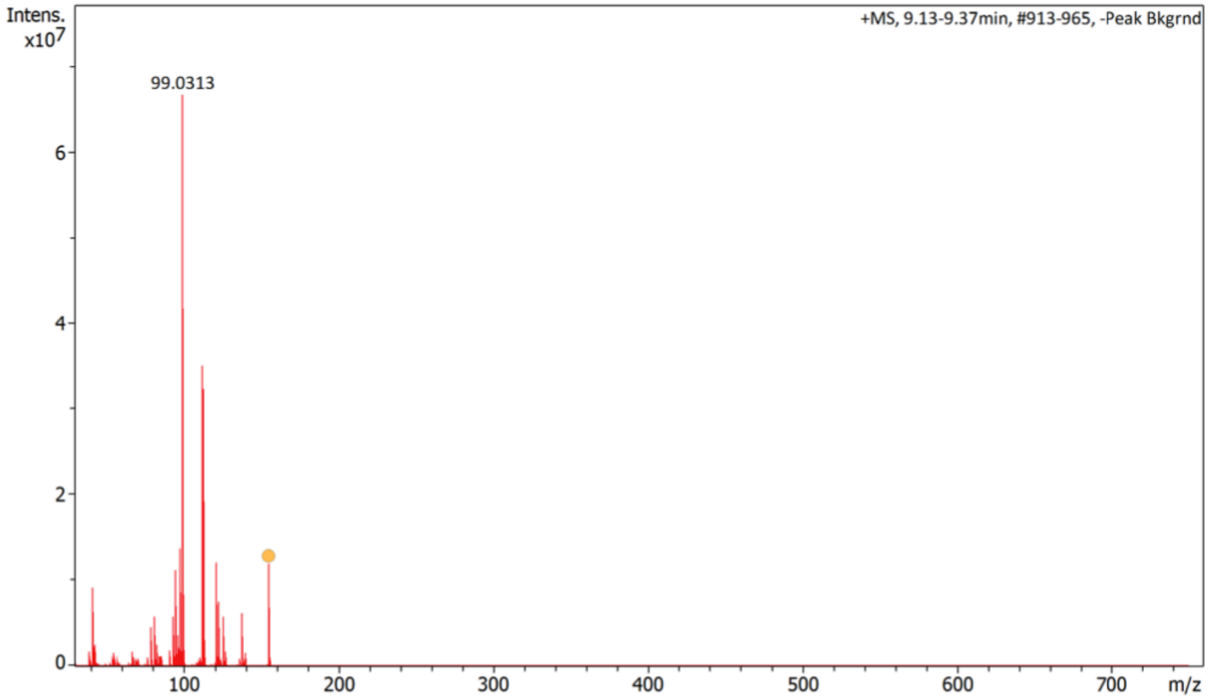

m/z	z	I	S/N	Res.
41.0384		9187951	132939.8	55973
42.0099		2261093	32715.6	53724
43.0416		2577460	37293.1	60370
43.0541		2156464	31201.7	54738
79.0542		4488383	64942.1	80206
81.0699		5758696	83322.2	63118
83.0491		2488155	36000.9	66908
93.0699		5801806	83945.9	67316
95.0855		11144994	161256.1	86422
96.0807		3571055	51669.3	80471
98.0237		13804596	199737.7	75466
99.0313		66679976	964787.9	74014
100.0347		3058431	44252.2	82682
112.0393		35023392	506751.0	72165
113.0470		32319436	467627.7	75302
121.0649		12123842	175419.0	76083
123.0805		7537965	109066.3	78265
126.0551		5728644	82887.3	78570
138.0915		6201723	89732.3	71869
155.0942		12070944	174653.6	73546

#	Ion Formula	Adduct	m/z	z	Meas. m/z	mSigma	N-Rule	err [mDa]	err [ppm]
1	C8H13NO2	M	155.0941	1+	155.0942	5.6	ok	-0.1	-0.7

Cmpd 10, 9.34 min

Supplementary Fig. 51. GC/MS spectrum of 5-pentylisoxazol-3-ol.

Supplementary Tables

Supplementary Table 1. Solubilizing agents used to prepare hydroxamic acid (HA) stock solutions assessed in the caecal content assay.

HA	Solubilizing agent
AHA	DMSO
HHA	HPβCD
OHA	HPβCD
NHA	HPβCD
DHA	HPβCD
LHA	MβCD
2-octynoHA	HPβCD
2-octenoHA	HPβCD
3-octenoHA	HPβCD
3-octynoHA	HPβCD/DMSO (90:10, v/v)
7-octynoHA	HPβCD/DMSO (90:10, v/v)

Note: The stock solutions of AHA were prepared in pure DMSO and were subsequently diluted in the assay to a final 1 % (v/v) DMSO. For HHA, OHA, NHA, DHA, 2-octynoHA, 2-octenoHA, 3-octenoHA, stock solutions were prepared using HPβCD at 1:4 mol/mol ratio, then for the assay serial dilutions were subsequently prepared from the stock solutions keeping the same ratio of molecules (1:4 mol/mol). For LHA, stock solutions were prepared using MβCD at 1:4 mol/mol ratio, then for the assay serial dilutions were prepared keeping the same ratio of molecules (1:4 mol/mol). For stock solutions of 3-octynoHA and 7-octynoHA, compounds were first dissolved in pure DMSO and then diluted with a solution of HPβCD to final 10% (v/v) DMSO and 1:3.6 (mol/mol) ratio of HA and HPβCD, respectively. In the assay, these stock solutions were subsequently diluted to 1% DMSO at the highest.

Supplementary Table 2. Calculated IC_{50} values for saturated and unsaturated aliphatic HAs. IC_{50} values are expressed as mean ± SD from *n* experiments. Source data are provided as a Source data file.

Compound	IC_{50} (mM)
Saturated aliphatic HAs	
AHA	8.67 ± 1.3 (3)
HHA	0.5 ± 0.2 (3)
OHA	0.25 ± 0.1 (3)
NHA	> 20
DHA	> 20
LHA	> 20
Unsaturated aliphatic HAs	
2-octynoHA	0.0093 ± 0.0089 (3)
2-octenoHA	0.28 ± 0.15 (3)
3-octenoHA	0.082 ± 0.034 (6)
3-octynoHA	0.11 ± 0.03 (3)
7-octynoHA	> 1

Note: The number of analyzed experimental replicates for each compound is shown in the brackets (n) next to the IC_{50} values.

Supplementary Table 3. Crystal data and structure refinement for 5-pentylisoxazol-3-ol.

CCDC No.	2257012
Empirical formula	$C_8H_{13}NO_2$
Formula weight	155.19
Temperature/K	100.0(1)
Crystal system	triclinic
Space group	*P*-1 (2)
a/Å	7.2549(4)
b/Å	7.5626(4)
c/Å	8.0022(5)
α/°	87.008(4)
β/°	77.410(5)
γ/°	75.682(4)
Volume/Å^3	415.18(4)
Z	2
ρ_{calc} g/cm^3	1.241
μ/mm^{-1}	0.089
F(000)	168
Crystal size/mm^3	0.476 × 0.153 × 0.044
Crystal colour	clear colourless
Crystal shape	plate
Radiation	Mo K_α (λ=0.71073)
2Θ range/°	5.93 to 69.41
Index ranges	-11 ≤ h ≤ 10, -12 ≤ k ≤ 10, -12 ≤ l ≤ 11
Reflections collected	9212
Independent reflections	3083 [R_{int} = 0.0329, R_{sigma} = 0.0391]
Data / Restraints / Param.	3083/1/104
Goodness-of-fit on F^2	1.055
Final *R* indexes [I≥2σ(I)]	R_1 = 0.0499, wR_2 = 0.1380
Final *R* indexes [all data]	R_1 = 0.0679, wR_2 = 0.1464
Largest peak/hole /eÅ^3	0.54/-0.26

Supplementary Table 4. Positive controls for the mutagenicity assay without S9 fraction.

Strain	Positive control	Concentration (µg/mL)
TA98	2-Nitrofluorene	2
TA100	4-Nitroquinoline-N-oxide	0.1
TA1535	N^4-Aminocytidine	100
TA1537	9-Aminoacridine	15
E. coli wp2 uvrA + E. coli wp2 [pKM101]	4-Nitroquinoline-N-oxide	2

Supplementary Table 5. Positive controls for the mutagenicity assay with S9 fraction.

Strain	Positive control	Concentration (µg/mL)
TA98	2-Aminoanthracene	0.5
TA100	2-Aminoanthracene	1.25
TA1535	2-Aminoanthracene	2.5
TA1537	2-Aminoanthracene	2.5
E. coli wp2 uvrA + E. coli wp2 [pKM101]	2-Aminofluorene	400

Supplementary Table 6. *In vitro* disintegration of coated gelatin capsules (size 0) in simulated gastric fluid (SGF, pH 1.2) and simulated intestinal fluid (SIF, pH 6.8) over time.

		Time in SGF (pH 1.2) (h)						Time in SIF (pH 6.8) (h)					
Coating	Layers	0.5	1	1.5	2	2.5	3	0.5	1	1.5	2	2.5	3
13% (w/w) Eudragit S100	2	12/12	12/12	11/12	11/12	11/12	3/12	2/12	2/12	1/12	1/12	1/12	0/12
	3	3/3	3/3	3/3	3/3	3/3	2/3	2/3	2/3	2/3	2/3	2/3	1/3
14% (w/w) Eudragit S100	2	7/7	7/7	7/7	7/7	7/7	5/7	5/7	3/7	2/7	1/7	1/7	1/7
	3	3/3	3/3	2/3	2/3	2/3	2/3	2/3	2/3	2/3	2/3	2/3	2/3
15% (w/w) Eudragit S100	1	3/3	3/3	3/3	3/3	3/3	0/3						
	2	8/8	8/8	8/8	8/8	8/8	8/8	8/8	8/8	7/8	7/8	5/8	2/8
	3	3/3	3/3	3/3	3/3	3/3	3/3	3/3	3/3	3/3	3/3	3/3	3/3
	4	3/3	3/3	3/3	3/3	3/3	3/3	3/3	3/3	3/3	3/3	3/3	3/3

Note: All tested coating solutions contained 5% (w/w) TEC as a plasticizer (see method section). Capsules were coated with various number of coating layers indicated in the column "Layers". Disintegration of capsules was monitored visually every 30 min for 3 h in both SGF and SIF. Coating performance was characterized by a number of capsules out of the overall amount of tested capsules with a corresponding coating that withstand incubation in SGF and SIF after every 30 min interval. The decrease in capsules number by at least 50% is indicated in red.

Supplementary Table 7. *In vitro* disintegration of coated gelatin capsules (size 0) in SGF (pH 1.2) and 50 mM KH_2PO_4 buffer (pH 5.5) over time.

		Time in SGF (pH 1.2) (h)						Time in 50 mM KH_2PO_4 buffer (pH 5.5) (h)					
Coating	Layers	0.5	1	1.5	2	2.5	3	0.5	1	1.5	2	2.5	3
13% (w/w) Eudragit S100	2	3/3	3/3	3/3	2/3	2/3	2/3	2/3	2/3	2/3	1/3	1/3	0/3
13% (w/w) Eudragit L100	2	3/3	3/3	3/3	1/3	1/3	1/3	0/3					
15% (w/w) Eudragit L100	2	3/3	3/3	3/3	3/3	3/3	3/3	2/3	1/3	0/3			
13% (w/w) Eudragit L100-55	2	3/3	3/3	3/3	0/3								
15% (w/w) Eudragit L100-55	2	3/3	3/3	3/3	0/3								

Note: All tested coating solutions contained 5% (w/w) TEC as a plasticizer (see method section). Capsules were coated with two coating layers indicated in the column "Layers". Disintegration of capsules was monitored visually every 30 min for 3 h in both SGF and phosphate buffer. Coating performance was characterized by a number of capsules out of the overall amount of tested capsules with corresponding coating that withstand incubation in SGF and phosphate buffer after every 30 min interval. The decrease in capsules number by at least 50% is indicated in red.

Supplementary Table 8. Summary of performed PK studies.

Study	Treatment	Dose of 2-octynoHA per dog (mg)	Route of administration	Blood collection time points
PK 1	Sterile solution of 2-octynoHA with HPβCD (1:1 mol/mol)	100	I.V.	Pre-dose, 5 min, 0.25, 0.5, 1, 1.5, 2.5, 4, 6, 8 and 24 h post-dose
PK 2	2-octynoHA in a gelatin capsule (size 0)	300	P.O.	Pre-dose, 0.25, 0.5, 1, 2, 4, 6, 12 h post-dose
PK 3	2-octynoHA in a coated gelatin capsule (size 0)	300	P.O.	Pre-dose, 0.5, 1, 2, 4, 6, 8, 12 and 24 h post-dose

Supplementary Table 9. Mass-over-charge ratio (*m/z*) and retention time of 2-octynoHA and its metabolites detected in PK 2 and PK 3.

Compound	*m/z*	Retention time (min)
2-octynoHA	156.1019	5.51
S12	262.1110	4.49
S22	448.1750	4.83
S32	156.1019	6.93
S33	332.1340	5.19

Supplementary Table 10. Description of PK parameters calculated by non-compartmental analysis.

Parameter	Definition
$t_{1/2}$	Half-life
t_{max}	Time to the maximum plasma concentration
C_{max}	Maximum observed plasma concentration
AUC_{0-t}	Area under the concentration-time curve from zero to the last measurable concentration

Supplementary Table 11. Validation of the analytical LC-UV method for quantification of 2-octynoHA and 5-pentylisoxazol-3-ol in the stability assay (retention time (RT), quality control (QC), precision (RSD)). Source data are provided as a Source data file.

Analyte	RT (min)	QC (mM)	Mean calculated concentration (mM) (*n*=3)	Bias (%)	RSD (%)
2-octynoHA	5.4	0.008	0.0086	7.2	4.41
		0.08	0.0843	5.4	2.46
		1	1.0637	6.4	2.43
5-pentylisoxazol-3-ol	6.5	0.04	0.0366	- 8.6	13.4
		0.08	0.0757	- 5.4	4.6
		1	1.0438	4.4	2.3

Supplementary Table 12. Validation of the LC-MS method for quantification of 2-octynoHA and 5-pentylisoxazol-3-ol in the permeability assay (retention time (RT), quality control (QC), intra-day precision (RSD_R), inter-day precision (RSD_T)). Source data are provided as a Source data file.

Analyte	RT (min)	QC (mM)	Mean calculated concentration (mM) (*n*=8)	Bias (%)	RSD_R (%)	RSD_T (%)
2-octynoHA	6.6	0.003	0.00323	7.8	5.0	13.7
		0.09	0.0999	11.0	5.65	13.74
5-pentylisoxazol-3-ol	8.7	0.003	0.00302	0.8	3.5	10.4
		0.09	0.09515	5.7	12.5	12.5

Supplementary Methods

Materials

Acetohydroxamic acid (AHA), 2-methyl-octynoate, 1,1'-carbonyldiimidazole (CDI), lithium acetylide ethylenediamine complex, 6-bromohexanoic acid, N-hydroxysuccinimide (NHS), iodobenzene diacetate, ethyl decanoate, hydroxylamine hydrochloride, hydroxylamine solution (50% wt. in H_2O), propylphosphonic anhydride solution (PPAA, 50% in ethyl acetate), potassium hydroxide (KOH), potassium phosphate monobasic (KH_2PO_4), urease from *Canavalia ensiformis* (Jack bean) type III (25,920 units/g solid), phosphoric acid (H_3PO_4), lucifer yellow CH dipotassium salt (LY), (Hydroxypropyl)methyl cellulose (average M_n ~ 86,000) (HPMC), neotame, Evans blue, methanol (MeOH, for HPLC, gradient grade) were purchased from Sigma-Aldrich. Methyl *trans*-2-octenoate, *trans*-3-octenoic acid and N-nitrosodiethylamine (DEN) were purchased from Tokyo Chemical Industry Co., Ltd. Oxalyl chloride and heptanol were purchased from abcr GmbH. Triethylamine, hydrogen peroxide (H_2O_2), triethyl citrate (TEC), tetrahydrofuran (THF, extra dry), MeOH (extra dry), acetonitrile (ACN, extra dry) were purchased from Acros Organics. ACN (LC/MS grade), ACN (Optima LC/MS grade), MeOH (Optima LC/MS grade) were obtained from Fisher Scientific. Dichloromethane (DCM, HPLC grade) and dimethyl sulfoxide (DMSO, extra dry) were purchased from Thermo Scientific. Lauraldehyde and potassium cyanide (KCN) were purchased from Merck Millipore. Nonanoic acid was purchased from Supelco. Octanohydroxamic acid (OHA) and oct-3-yn-1-ol were obtained from Tokyo Chemical Industry Co., Ltd and abcr GmbH. Methyl-β-cyclodextrin (DS∼12) (MβCD) and (2-hydroxypropyl)-β-cyclodextrin (DS∼4.5) (HPβCD) were purchased from CycloLab. Urea and sodium hydroxide (NaOH) were purchased from VWR Chemicals. Eudragit S100, Eudragit L100 and Eudragit L100-55 were purchased from Evonik. Rifaximin was obtained from Nanjing Cuccess Pharmaceutical Co. Mannitol was obtained from Pharmatrans Sanaq AG. Formic acid was purchased from Sigma-Aldrich and Thermo Fisher Scientific. Phenol red was purchased from Fluka. All chemicals were used without additional purification.

Synthesis of 2-octynohydroxamic acid

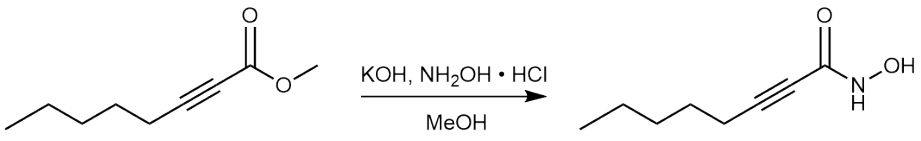

KOH (0.74 g, 13.0 mmol) and hydroxylamine hydrochloride (0.90 g, 13.0 mmol) were dissolved in 3 and 6 mL of methanol (MeOH), respectively. The KOH solution was added to the hydroxylamine hydrochloride solution with stirring under N_2 atmosphere on ice, whereupon a white precipitate (KCl) was observed. Once all the KOH had been added, the mixture was allowed to stir for 20 min to ensure complete precipitation of KCl. The mixture was filtered under vacuum, and methyl 2-octynoate (1.0 g, 6.50 mmol) was added to the filtrate. After stirring at room temperature (RT) for 24 h, the solution was extracted with dichloromethane (DCM) (20 mL) and washed with brine (20 mL). The solution was dried and concentrated under vacuum. The obtained crude product was mixed with celite, loaded in a solid load cartridge and then purified by medium pressure liquid chromatography (MPLC) system (Combi*Flash*® NextGen 300+, Teledyne ISCO) (DCM/MeOH = 100/0 to 80/20, v/v) to give 0.039 g of white pinkish 2-octynohydroxamic acid solid (2-octynoHA) (yield: 4%).

^{1}H NMR (400 MHz, DMSO-d6) δ 10.97 (s, 1H), 9.09 (s, 1H), 2.33 (t, *J* = 7.0 Hz, 2H), 1.52 - 1.45 (m, 2H), 1.40 – 1.22 (m, 4H), 0.88 (t, *J* = 7.2 Hz, 3H) (Supplementary Fig. 23).

^{13}C NMR (100 MHz, DMSO-d6) δ 150.8, 88.3, 74.4, 30.8, 27.5, 22.1, 18.2, 14.3 (Supplementary Fig. 24).

HRMS (ESI) *m/z* calculated for $C_8H_{14}NO_2$ $[M+H]^+$ 156.1019, found 156.1018 (Supplementary Fig. 25).

Synthesis of *trans*-2-octenohydroxamic acid

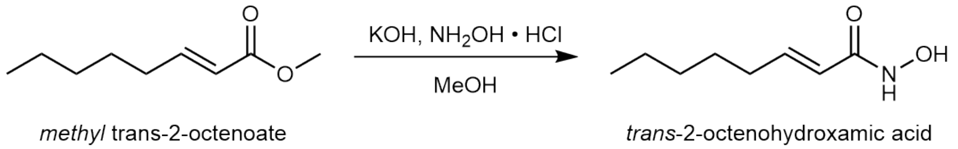

KOH (2.15 g, 38.41 mmol) and hydroxylamine hydrochloride (1.33 g, 19.20 mmol) and were separately prepared in 10 mL of MeOH and were placed on ice. The solution of KOH was added to the hydroxylamine hydrochloride solution. After stirring for 20-30 min, methyl *trans*-2-octenoate (0.30 g, 1.92 mmol) in a small amount of MeOH was added to the mixture in ice bath, and the mixture was stirred for 5 min. Then, the reaction was continued at RT for 24 h, the mixture was diluted in 50 mL water (H_2O) and acidified with 2 M HCl. The mixture was extracted with DCM (3 x 50 mL), dried over $MgSO_4$, and concentrated under vacuum. The product was purified by column chromatography (DCM/MeOH = 98/2 to 90/10, v/v) to obtain 0.10 g of *trans*-2-octenohydroxamic acid (2-octenoHA) (yield: 33%).

^{1}H NMR (400 MHz, DMSO-d6) δ 10.51 (s, 1H), 8.83 (s, 1H), 6.66 – 6.59 (m, 1H), 5.73 (d, *J* = 15.6 Hz, 1H), 2.15 – 2.10 (m, 2H), 1.46 – 1.19 (m, 6H), 0.87 (t, *J* = 7.0 Hz, 3H) (Supplementary Fig. 26).

^{13}C NMR (100 MHz, DMSO-d6) δ 163.2, 142.7, 121.7, 31.7, 31.2, 28.0, 22.4, 14.3 (Supplementary Fig. 27).

HRMS (ESI) *m/z* calculated for $C_8H_{16}NO_2$ $[M+H]^+$ 158.1176, found 158.1179 (Supplementary Fig. 28).

Synthesis of trans-3-octenohydroxamic acid

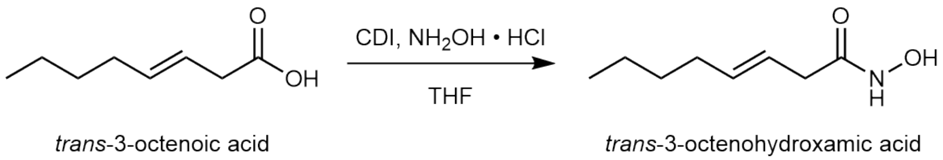

CDI (0.86 g, 5.27 mmol) was added to 5 mL dry tetrahydrofuran (THF) solution of *trans*-3-octenoic acid (0.50 g, 3.52 mmol) and stirred for 1 h.[9] Then, hydroxylamine hydrochloride (0.50 g,

7.03 mmol) was added to the mixture and left to stir overnight. The reaction was quenched with 5% aq. $KHSO_4$ (30 mL) and extracted with DCM (2 × 30 mL). The combined organic phases were washed with brine (30 mL) and dried over Na_2SO_4. The extract was filtered, concentrated under vacuum and purified using the MPLC system (DCM/MeOH = 100/0 to 80/20, v/v) to give 0.205 g of *trans*-3-octenohydroxamic acid (3-octenoHA) as yellowish crystals (yield: 37%).

^{1}H NMR (400 MHz, DMSO-d6) δ 10.37 (s, 1H), 8.70 (s, 1H), 5.56 – 5.36 (m, 2H), 2.67 (d, *J* = 5.6 Hz, 2H), 1.99 - 1.94 (m, 2H), 1.34 – 1.21 (m, 4H), 0.90 – 0.82 (t, *J* = 7.2 Hz, 3H) (Supplementary Fig. 29).

^{13}C NMR (100 MHz, DMSO-d6) δ 167.8, 133.4, 124.1, 37.0, 32.0, 31.4, 22.1, 14.3 (Supplementary Fig. 30).

HRMS (ESI) *m/z* calculated for $C_8H_{16}NO_2$ $[M+H]^+$ 158.1176, found 158.1181 (Supplementary Fig. 31).

Synthesis of 3-octynohydroxamic acid

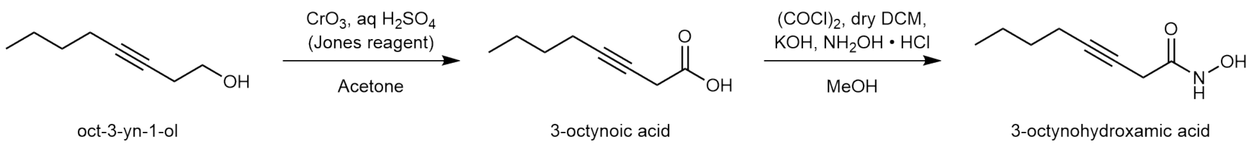

Oct-3-yn-1-ol (1.13 mL, 7.92 mmol) was dissolved in acetone (20 mL) and cooled to 0 °C in an ice bath. Jones reagent (15 mL, 2.13 M, 32 mmol) was added dropwise, after which a dark precipitate was observed. After stirring at 0 °C for 15 min, the reaction was quenched by the dropwise addition of 2-propanol (8 mL). After stirring at 0 °C for an additional 15 min, the mixture turned colorless and the blue precipitate was formed. The mixture was diluted with H_2O (30 mL) and subsequently extracted with diethyl ether (4 x 25 mL). The combined organic phases were filtered through celite, washed with 10% (w/v) $Na_2S_2O_3$ (4 x 100 mL), saturated Na_2CO_3 (3 x 100 mL), and NaCl (3 x 100 mL) and lastly dried over Na_2SO_4. The solvent was evaporated under vacuum to obtain yellow oil of 3-octynoic acid (0.45 g, yield: 40.5%), which was used in the following reaction without further purification.

Oxalyl chloride ($(COCl)_2$) (0.31 mL, 3.57 mmol) was added to 4.2 mL dry DCM solution of 3-octynoic acid (0.20 g, 1.43 mmol). The reaction was stirred at RT for 1 h. The solvent was evaporated under vacuum. Solutions of hydroxylamine hydrochloride (0.21 g, 2.86 mmol) in 3 mL MeOH, KOH (0.21 g, 2.86 mmol) in 3 mL MeOH and 200 μL hydroxylamine solution (50% wt. in H_2O) were added to the residue. The mixture was stirred at RT for 1 h, whereupon a white precipitate was observed. Then, the mixture was acidified with 0.1 M HCl and extracted with DCM (3 x 10 mL). The combined organic phases were washed with brine, dried over $MgSO_4$ and concentrated under vacuum. The residue was purified using the MPLC system (DCM/MeOH = 100/0 to 90/10, v/v) to give 0.0297 g of 3-octynohydroxamic acid (3-octynoHA) (yield: 13.4%).

^{1}H NMR (400 MHz, DMSO-d6) δ 10.48 (s, 1H), 8.89 (s, 1H), 2.92 (t, J = 2.4 Hz, 2H), 2.17 - 2.13 (m, 2H), 1.46 – 1.29 (m, 4H), 0.87 (t, J = 7.2 Hz, 3H) (Supplementary Fig. 32).

^{13}C NMR (100 MHz, DMSO-d6) δ 164.4, 82.6, 74.5, 30.8, 24.5, 21.8, 18.2, 13.9 (Supplementary Fig. 33).

HRMS (ESI) *m/z* calculated for $C_8H_{14}NO_2$ $[M+H]^+$ 156.1019, found 156.1023 (Supplementary Fig. 34).

Synthesis of 7-octynohydroxamic acid

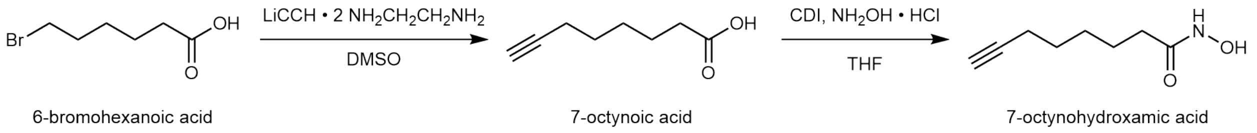

7-octynohydroxamic acid (7-octynoHA) was synthesized from 7-octynoic acid, which was produced *via* the SN2 alkylation using a lithium acetylide ethylenediamine complex and 6-bromohexanoic acid. In a flame dried flask, lithium acetylide ethylenediamine complex (0.69 g, 7.69 mmol) was dissolved in anhydrous DMSO and cooled to 0°C. Then, 5 mL DMSO solution of 6-bromohexanoic acid (1.0 g, 5.13 mmol) were added. After stirring for 3 h, the reaction was quenched on ice with brine, acidified with 2 M HCl, and extracted with DCM (3 x 50 mL). The combined organic phases were dried over $MgSO_4$. The solvent was evaporated under vacuum to

obtain colorless oil of 7-octynoic acid (0.65 g, yield: 100%), which was used in the next reaction without further purification.

7-octynoic acid (0.90 g, 6.42 mmol) obtained in the previous step was dissolved in THF. Then, CDI (3.12 g, 19.26 mmol) was added to the solution, and the mixture was stirred vigorously for 1 - 2 h. After that, hydroxylamine hydrochloride (2.23 g, 32.10 mmol) was added. After stirring at RT for 20 h, the reaction was quenched with $KHSO_4$ (30 mL) and extracted with DCM (3 x 50 mL). The combined organic phases were dried over $MgSO_4$ and concentrated under vacuum to obtain yellowish oil. The crude was purified by column chromatography (DCM/MeOH = 98/2 to 90/10, v/v). 7-octynoHA was obtained as white crystals (0.40 g, yield: 40%).

^{1}H NMR (400 MHz, DMSO-d6) δ 10.33 (s, 1H), 8.66 (s, 1H), 2.74 (t, *J* = 2.6 Hz, 1H), 2.16 - 2.12 (m, 2H), 1.94 (t, *J* = 7.2 Hz, 2H), 1.53 – 1.40 (m, 4H), 1.37 – 1.28 (m, 2H) (Supplementary Fig. 35).

^{13}C NMR (100 MHz, DMSO-d6) δ 168.9, 84.4, 71.1, 54.8, 32.1, 27.6, 24.5, 17.5 (Supplementary Fig. 36).

HRMS (ESI) *m/z* calculated for $C_8H_{14}NO_2$ $[M+H]^+$ 156.1019, found 156.1022 (Supplementary Fig. 37).

Synthesis of heptanohydroxamic acid

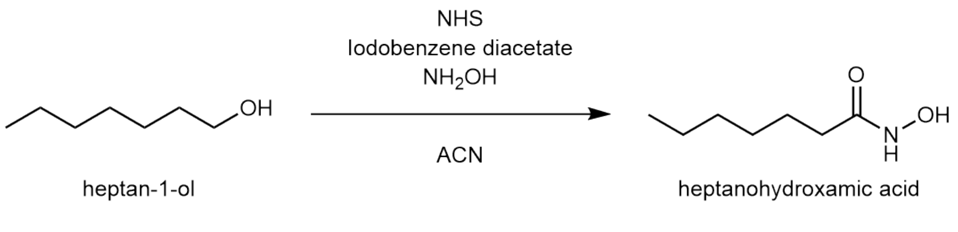

Heptan-1-ol (0.60 mL, 4.30 mmol) was added to a solution of NHS (0.54 g, 4.73 mmol) and iodobenzene diacetate (2.91 g, 9.04 mmol) in 5.5 mL acetonitrile (ACN) at 0 °C under argon.[10] After the addition of heptan-1-ol, the black suspension turned to a bright orange solution. The reaction mixture was stirred for 1 h at the same temperature until the disappearance of heptan-1-

ol, after which hydroxylamine solution (50% wt. in H_2O) (0.55 mL, 18.07 mmol) was added, resulting in the formation of a white precipitate. The reaction mixture was allowed to warm to RT and was stirred overnight. The solvent was removed under vacuum, and the residue was purified by the MPLC system (DCM/MeOH = 100/0 to 80/20, v/v). Heptanohydroxamic acid (HHA) was subsequently crystalized from hexane in 0.117 g (yield: 18.7 %).

^{1}H NMR (400 MHz, DMSO-d6) δ 10.33 (s, 1H), 8.71 (s, 1H), 1.93 (t, J = 7.6 Hz, 2H), 1.51 - 1.44 (m, 2H), 1.29 - 1.24 (m, 6H), 0.86 (t, J =6.8 Hz, 3H) (Supplementary Fig. 38).

^{13}C NMR (100 MHz, DMSO-d6) δ 169.6, 32.7, 31.4, 28.7, 25.7, 22.5, 14.4 (Supplementary Fig. 39).

HRMS (ESI) *m/z* calculated for $C_7H_{15}NNaO_2$ $[M+Na]^+$ 168.0995, found 168.0994 (Supplementary Fig. 40).

Synthesis of nonanonohydroxamic acid

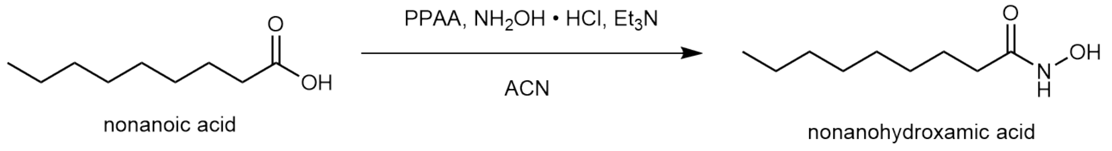

Triethylamine (Et_3N) (1.76 mL, 12.64 mmol) was added to 10 mL ACN (anh.) solution of PPAA (50% wt. in ethyl acetate, 2.20 mL, 7.58 mmol) obtaining a clear yellow solution. Nonanoic acid (0.56 mL, 3.16 mmol) was added, resulting in the formation of a clear orange colour.[11] After stirring for 30 min at RT, hydroxylamine hydrochloride (0.45 g, 6.32 mmol) was added. After stirring overnight at RT, the reaction was extracted with DCM (30 mL), washed with brine (2 x 30 mL) and dried over Na_2SO_4 for 30 min. The solvent was evaporated, the residue was purified by the MPLC system (DCM/MeOH = 100/0 to 80/20, v/v) to give 0.201 g of nonanohydroxamic acid (NHA) (yield: 36.7%).

^{1}H NMR (400 MHz, DMSO-d6) δ 10.31 (s, 1H), 8.66 (s, 1H), 1.93 (t, J = 7.4 Hz, 2H), 1.51 - 1.46 (m, 2H), 1.30 - 1.24 (m, 10H), 0.86 (t, J = 7.0 Hz, 3H) (Supplementary Fig. 41).

^{13}C NMR (100 MHz, MeOD) δ 171.7, 32.4, 31.6, 29.0, 28.9, 28.8, 25.4, 22.3, 13.0 (Supplementary Fig. 42).

HRMS (ESI) *m/z* calculated for $C_9H_{20}NO_2$ $[M+H]^+$ 174.1489, found 174.1489 (Supplementary Fig. 43).

Synthesis of decanohydroxamic acid

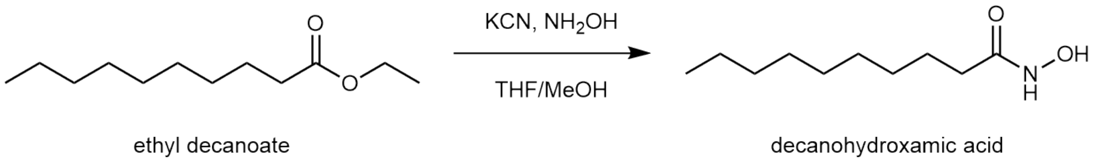

Ethyl decanoate (0.58 mL, 2.50 mmol) was dissolved in 4 mL THF/MeOH (1:1 v/v). Hydroxylamine solution (50% wt. in H_2O) (1.10 mL, 3.50 mmol) and KCN (16.8 mg, 0.25 mmol) were added subsequently. The reaction mixture was stirred at RT overnight after which the starting material was not detected. The reaction was worked up with brine (10 mL), extracted with DCM (3 x 10 mL), dried over Na_2SO_4 for 30 min and evaporated. Decanohydroxamic acid (DHA) was obtained as a white solid without further purification (0.201 g, yield: 43%).

^1H NMR (400 MHz, DMSO-d6) δ 9.51 (s, 1H), 1.92 (t, J = 7.4 Hz, 2H), 1.50 - 1.43 (m, 2H), 1.29 - 1.23 (m, 12H), 0.85 (t, J = 6.8 Hz, 3H) (Supplementary Fig. 44).

^{13}C NMR (100 MHz, DMSO-d6) δ 169.5, 32.7, 31.8, 29.4, 29.2, 29.2, 29.1, 25.6, 22.6, 14.4 (Supplementary Fig. 45).

HRMS (ESI) *m/z* calculated for $C_{10}H_{22}NO_2$ $[M+H]^+$ 188.1645, found 188.1648 (Supplementary Fig. 46).

Synthesis of laurohydroxamic acid

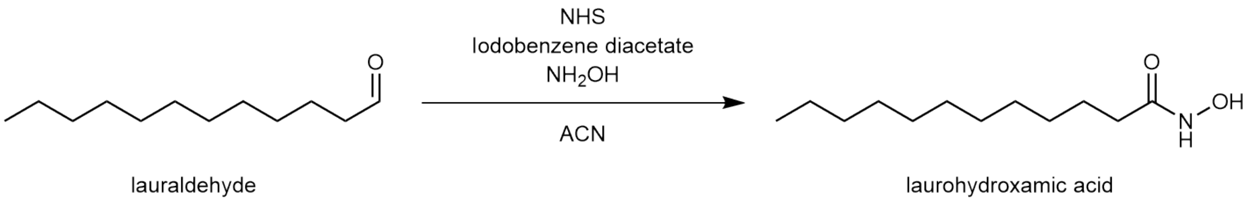

Lauraldehyde (2.50 mL, 10.85 mmol) was added to 18 mL ACN solution of NHS (1.37 g, 11.94 mmol) and iodobenzene diacetate (3.91 g, 11.94 mmol) at 0 °C under argon.[12] After the addition of the aldehyde, the black suspension solution turned to a bright orange solution. The reaction mixture was stirred for 1 h at 0 °C, and was monitored by TLC until lauraldehyde disappears. Then, hydroxylamine solution (50% wt. in H_2O) (1.40 mL, 43.40 mmol) was added, resulting in the formation of a white precipitate. The reaction mixture was allowed to warm to RT and was stirred overnight. The solvent was removed under vacuum, and the residue was purified by the MPLC system (DCM/MeOH = 100/0 to 55/45, v/v) to obtain 0.103 g of laurohydroxamic acid (LHA) (yield: 4.4%).

^{1}H NMR (400 MHz, DMSO-d6) δ 10.31 (s, 1H), 8.64 (s, 1H), 1.92 (t, J = 7.4 Hz, 2H) 1.50 - 1.43 (m, 2H), 1.29 - 1.19 (m, 16H), 0.85 (t, J = 7.0 Hz, 3H) (Supplementary Fig. 47).

^{13}C NMR (100 MHz, DMSO-d6) δ 169.6, 32.7, 31.8, 29.5, 29.4, 29.2, 29.1, 25.6, 22.6, 14.4 (Supplementary Fig. 48).

HRMS (ESI) *m/z* calculated for $C_{12}H_{26}NO_2$ $[M+H]^+$ 216.1958, found 216.1958 (Supplementary Fig. 49).

Production of 5-pentylisoxazol-3-ol

Eighteen milliliters of 10 mM 2-octynoHA (28 mg) in 200 mM KH_2PO_4 (pH 6.8) were incubated overnight at 37 °C with shaking after which the starting material was not detected. The reaction was extracted with chloroform (18 mL), dried over Na_2SO_4 and concentrated under vacuum. The product was obtained as a yellowish solid without further purification (17.4 mg, yield: 62%).

^{1}H NMR (400 MHz, DMSO-d6) δ 11.00 (s, 1H), 5.75 (s, 1H), 2.59 (t, J = 7.8 Hz, 2H) 1.62 - 1.54 (m, 2H), 1.31 - 1.27 (m, 4H), 0.87 (t, J = 7.0 Hz, 3H) (Supplementary Fig. 50).

GC/MS *m/z* calculated for $C_8H_{13}NO_2$ $[M]^+$ 155.0941, found 155.0942 (Supplementary Fig. 51).

Supplementary References

1. Begum, A., Choudhary, M. I. & Betzel, C. The first Jack bean urease (Canavalia ensiformis) complex obtained at 1.52 resolution. https://doi.org/10.2210/pdb4h9m/pdb (2012).

2. Benini, S., Rypniewski, W. R., Wilson, K. S., Ciurli, S. & Mangani, S. Structure of Bacillus Pasteurii urease inhibited with acetohydroxamic acid at 1.55 A resolution. https://doi.org/10.2210/pdb4ubp/pdb (1999).

3. Benini, S. *et al.* The complex of Bacillus pasteurii urease with acetohydroxamate anion from X-ray data at 1.55 Å resolution. *JBIC J. Biol. Inorg. Chem.* **5**, 110–118 (2000).

4. Ha, N.-C. *et al.* Supramolecular assembly and acid resistance of Helicobacter pylori urease. *Nat. Struct. Biol.* **8**, 505–509 (2001).

5. Ha, N.-C., Oh, S.-T. & Oh, B.-H. Crystal structure of Helicobacter pylori urease in complex with acetohydroxamic acid. https://doi.org/10.2210/pdb1e9y/pdb (2000).

6. Pearson, M. A., Michel, L. O., Hausinger, R. P. & Karplus, P. A. Structures of Cys319 Variants and Acetohydroxamate-Inhibited Klebsiella aerogenes Urease,. *Biochemistry* **36**, 8164–8172 (1997).

7. Pearson, M. A. & Karplus, P. A. Klebsiella Aerogenes urease, C319A variant with acetohydroxamic acid (AHA) bound. http://doi.org/10.2210/pdb1fwe/pdb (1997).

8. Humphrey, W., Dalke, A. & Schulten, K. VMD: visual molecular dynamics. *J. Mol. Graph.* **14**, 33–38, 27–28 (1996).

9. Usachova, N., Leitis, G., Jirgensons, A. & Kalvinsh, I. Synthesis of hydroxamic acids by activation of carboxylic acids with N,N′-carbonyldiimidazole: exploring the efficiency of the method. *Synth. Commun.* **40**, 927–935 (2010).

10. Dettori, G., Gaspa, S., Porcheddu, A. & Luca, L. D. A two-step tandem reaction to prepare hydroxamic acids directly from alcohols. *Org. Biomol. Chem.* **12**, 4582–4585 (2014).

11. Ech-Chahad, A., Minassi, A., Berton, L. & Appendino, G. An expeditious hydroxyamidation of carboxylic acids. *Tetrahedron Lett.* **46**, 5113–5115 (2005).

12. Dettori, G., Gaspa, S., Porcheddu, A. & De Luca, L. One-pot synthesis of hydroxamic acids from aldehydes and hydroxylamine. *Adv. Synth. Catal.* **356**, 2709–2713 (2014).
