# Supplementary material for: Inhibition of urease-mediated ammonia production by 2-octynohydroxamic acid in hepatic encephalopathy
Source: Nat Commun. 2024 Mar 12;15:2226. doi: 10.1038/s41467-024-46481-8 (PMC10933438; doi:10.1038/s41467-024-46481-8)
Supplement: Supplementary file 3 — Description of Additional Supplementary Files [file 41467_2024_46481_MOESM3_ESM.pdf]

<u>Description of Additional Supplementary Files</u>

Supplementary Datasets

Supplementary Data 1. P-values calculated by one-way ANOVA with Tukey's multiple comparisons test for the cell viability in the presence of 2-octynoHA experiments.

Supplementary Data 2. P-values calculated by two-way repeated measures ANOVA with Tukey's multiple comparisons test for ammonia levels in DEN rats.

Supplementary Data 3. P-values calculated by one-way repeated measures ANOVA with Tukey's multiple comparisons test for ammonia levels in BDL rats.

Supplementary Data 4. P-values calculated by one-way ANOVA with Tukey's multiple comparisons test for brain glutamine (Gln) levels in BDL rats.

Supplementary Data 5. P-values calculated by one-way ANOVA with Tukey's multiple comparisons test for brain myo-inositol (Ins) levels in BDL rats.

Supplementary Data 6. P-values calculated by one-way ANOVA with Tukey's multiple comparisons test for brain taurine (Tau) levels in BDL rats.

Supplementary Data 7. P-values calculated by one-way ANOVA with Tukey's multiple comparisons test for brain creatine (Cr) levels in BDL rats.

Supplementary Data 8. P-values calculated by one-way ANOVA with Tukey's multiple comparisons test for brain phosphocreatine (PCr) levels in BDL rats.

Supplementary Data 9. P-values calculated by one-way ANOVA with Tukey's multiple comparisons test for brain total creatine (tCr) levels in BDL rats.

Supplementary Data 10. P-values calculated by one-way ANOVA with Tukey's multiple comparisons test for brain total choline (tCh) levels in BDL rats.

Supplementary Data 11. P-values calculated by one-way ANOVA with Tukey's multiple comparisons test for brain glutamate (Glu) levels in BDL rats.

Supplementary Data 12. P-values calculated by one-way ANOVA with Tukey's multiple comparisons test for brain γ-aminobutyric acid (GABA) levels in BDL rats.

Supplementary Data 13. P-values calculated by one-way ANOVA with Tukey's multiple comparisons test for the cell viability in the presence of 5-pentylisoxazol-3-ol experiments.

Supplementary Data 14. P-values calculated by one-way ANOVA with Tukey's multiple comparisons test for the cell viability in the presence of octanohydroxamic acid (OHA) experiments.

Supplementary Data 15. P-values calculated by one-way ANOVA with Tukey's multiple comparisons test for the cell viability in the presence of acetohydroxamic acid (AHA) experiments.

Supplementary Data 16. P-values calculated by one-way repeated measured ANOVA with Tukey's multiple comparisons test for body weight of DEN rats.

Supplementary Data 17. P-values for comparison of bilirubin levels between groups of BDL rats was calculated by two-way repeated measured ANOVA with Tukey's multiple comparisons test. P-values for comparison of bilirubin levels between treatment days within a group of BDL rats was calculated by one-way repeated measured ANOVA with Tukey's multiple comparisons test.

Supplementary Data 18. P-values for comparison of body weight between treatment days within a group of BDL rats was calculated by one-way repeated measured ANOVA with Tukey's multiple comparisons test.

Supplementary Data 19. P-values for comparison of glucose levels between groups of BDL rats was calculated by two-way repeated measured ANOVA with Tukey's multiple comparisons test. P-values for comparison of glucose levels between treatment days within a group of BDL rats was calculated by one-way repeated measured ANOVA with Tukey's multiple comparisons test.

Supplementary Data 20. P-values calculated by one-way ANOVA with Tukey's multiple comparisons test for brain glucose (Glc) levels in BDL rats.

Supplementary Data 21. P-values calculated by one-way ANOVA with Tukey's multiple comparisons test for brain ascorbate (Asc) levels in BDL rats.

Supplementary Data 22. P-values calculated by one-way ANOVA with Tukey's multiple comparisons test for brain lactate (Lac) levels in BDL rats.

Supplementary Data 23. P-values calculated by one-way ANOVA with Tukey's multiple comparisons test for brain Nacetylaspartylglutamate (NAAG) levels in BDL rats.

Supplementary Data 24. P-values calculated by one-way ANOVA with Tukey's multiple comparisons test for brain phosphoethanolamine (PE) levels in BDL rats.

Supplementary Data 25. P-values calculated by one-way ANOVA with Tukey's multiple comparisons test for brain N-acetylaspartate (NAA) levels in BDL rats.
