# Supplementary material for: Inhibition of urease-mediated ammonia production by 2-octynohydroxamic acid in hepatic encephalopathy
Source: Nat Commun. 2024 Mar 12;15:2226. doi: 10.1038/s41467-024-46481-8 (PMC10933438; doi:10.1038/s41467-024-46481-8)
Supplement: Supplementary file 5 — Reporting Summary [file 41467_2024_46481_MOESM5_ESM.pdf]

Corresponding author(s): Jean-Christophe Leroux
Last updated by author(s): Jan 24, 2024

Reporting Summary

Nature Portfolio wishes to improve the reproducibility of the work that we publish. This form provides structure for consistency and transparency in reporting. For further information on Nature Portfolio policies, see our Editorial Policies and the Editorial Policy Checklist.

Statistics

For all statistical analyses, confirm that the following items are present in the figure legend, table legend, main text, or Methods section.

n/a	Confirmed	
☐	☒	The exact sample size (*n*) for each experimental group/condition, given as a discrete number and unit of measurement
☐	☒	A statement on whether measurements were taken from distinct samples or whether the same sample was measured repeatedly
☐	☒	The statistical test(s) used AND whether they are one- or two-sided<br>*Only common tests should be described solely by name; describe more complex techniques in the Methods section.*
☒	☐	A description of all covariates tested
☐	☒	A description of any assumptions or corrections, such as tests of normality and adjustment for multiple comparisons
☐	☒	A full description of the statistical parameters including central tendency (e.g. means) or other basic estimates (e.g. regression coefficient) AND variation (e.g. standard deviation) or associated estimates of uncertainty (e.g. confidence intervals)
☐	☒	For null hypothesis testing, the test statistic (e.g. *F*, *t*, *r*) with confidence intervals, effect sizes, degrees of freedom and *P* value noted<br>*Give P values as exact values whenever suitable.*
☒	☐	For Bayesian analysis, information on the choice of priors and Markov chain Monte Carlo settings
☒	☐	For hierarchical and complex designs, identification of the appropriate level for tests and full reporting of outcomes
☒	☐	Estimates of effect sizes (e.g. Cohen's *d*, Pearson's *r*), indicating how they were calculated

Our web collection on statistics for biologists contains articles on many of the points above.

Software and code

Policy information about availability of computer code

Data collection	CrysAlisPro was used to collect and process crystallography data. Tecan i-control 2.0.10.0 and Microsoft Excel were used to collect and process data generated by a plate reader (i.e. caecal content-based screening assay, pH-based cell-free urease activity assay, cytotoxicity, Lucifer Yellow fluorescence). Chromeleon 7.2.9 was used to collect and process LC-UV/Vis data from the stability test. Collection and processing of mass spectrometry (MS) data from the permeability assay was done using Xcalibur software (Version 3.3.2 SP2). MS data from the PK studies were processed using UNIFI 1.9.4, Waters Connect 2.2 and UNIFI 2.1.2.14 softwares. MRS spectra were processed using LCModel.
Data analysis	Statistical and regression analyses were performed using GraphPad Prism 9.4.1, GraphPad Prism 8.2, and Microsoft Excel. PK parameters were calculated using PKSolver 2.0 add-in for Microsoft Excel.

For manuscripts utilizing custom algorithms or software that are central to the research but not yet described in published literature, software must be made available to editors and reviewers. We strongly encourage code deposition in a community repository (e.g. GitHub). See the Nature Portfolio guidelines for submitting code & software for further information.

Data

Policy information about availability of data

All manuscripts must include a data availability statement. This statement should provide the following information, where applicable:

- Accession codes, unique identifiers, or web links for publicly available datasets
- A description of any restrictions on data availability
- For clinical datasets or third party data, please ensure that the statement adheres to our policy

The urease structures described and visualized in the Introduction section were obtained from the Protein Data Bank (PDB) with accession codes 4H9M (https://doi.org/10.2210/pdb4h9m/pdb), 4UBP (https://doi.org/10.2210/pdb4ubp/pdb), 1E9Y (https://doi.org/10.2210/pdb1e9y/pdb), 1FWE (http://doi.org/10.2210/pdb1fwe/pdb). The data generated in the in vitro studies (caecal content-based and pH-based cell-free urease activity assays, cytotoxicity, mutagenicity, stability, permeability experiments), PK and in vivo studies in rats are provided in the Source Data file. Source data are provided with this paper. Results of statistical analysis are provided in the Supplementary Data file. Raw spectra data generated in in vivo 1H MRS experiments are available in Zenodo (https://doi.org/10.5281/zenodo.8042020). X-ray crystallographic data for 5-pentylisoxazol-3-ol have been deposited at the Cambridge Crystallographic Data Centre under deposition number 2257012 (https://dx.doi.org/1s0.5517/ccdc.csd.cc2frlvn). The remaining data generated in this study are available in the Supplementary Information.

Research involving human participants, their data, or biological material

Policy information about studies with human participants or human data. See also policy information about sex, gender (identity/presentation), and sexual orientation and race, ethnicity and racism.

Reporting on sex and gender	Not applicable
Reporting on race, ethnicity, or other socially relevant groupings	Not applicable
Population characteristics	Not applicable
Recruitment	Not applicable
Ethics oversight	Not applicable

Note that full information on the approval of the study protocol must also be provided in the manuscript.

Field-specific reporting

Please select the one below that is the best fit for your research. If you are not sure, read the appropriate sections before making your selection.

☒ Life sciences ☐ Behavioural & social sciences ☐ Ecological, evolutionary & environmental sciences

For a reference copy of the document with all sections, see nature.com/documents/nr-reporting-summary-flat.pdf

Life sciences study design

All studies must disclose on these points even when the disclosure is negative.

Sample size

No sample size calculation was performed.

In the in vivo studies in BDL rats, we used between 10 to 20 animals per group, with a total of 4 groups (55 animals). The group size was based on the previously published MRS measurements in BDL rats by a co-author. Dr. Cudalbu, where 7-14 animals per group were used (Simicic, D., Rackayova, V., Braissant, O. et al. Metab Brain Dis 38, 1999–2012 (2023); Rackayova V, Braissant O, Rougemont AL, Cudalbu C, McLin VA. Sci Rep. 2020 May 5;10(1):7536; Račkayová V, Simicic D, Donati G, et al. J Neurochem. 2021; 157: 508–519). Furthermore, the sample size was limited by the complexity, duration and number of groups for the MRS experiments, which in turn is limited by ethical concerns (e.g. the authorisation for the animal experimentation).

In DEN rats, 10 animals per group (4 groups and 40 animals in total) were used based on our previous experience with blood ammonia measurements in rats showing that such number of animals is sufficient to detect differences between groups.

For PK studies, the sample size (n=3 per group) was determined by available resources for subcontracted studies.

For in vitro experiments, the minimum sample size was set at n=3 as it is widely accepted in practice.

All sample sizes are provided in the figure legends.

Data exclusions

No data were excluded from the analysis.

Replication	To verify the reproducibility of the experimental findings, the experiments were performed at least in triplicate (in the case of in vitro tests) or at least three animals per group were used (in the case of in vivo studies). All attempts at replication were successful.
Randomization	In most in vivo studies, only one group was examined at a time, and randomization was not needed. In the studies with BDL rats, the negative control group and the group treated with 2-octynoHA suspension were studied in parallel. In these cases, each group contained up to 4 animals, and the rats were divided into two groups on day 35 post-surgery to achieve similar mean blood ammonia levels in both groups prior to the first treatment administration.<br>In the DEN model studies, a negative control group and a group treated with rifaximin 30 mg/kg were studied in parallel. In these cases, each group contained 5 animals, rats were divided into two groups to achieve similar mean blood ammonia levels in both groups at the start of the study. Two other groups (rifaximin 60 mg/kg and 2-octynoHA) were studied individually and were separated in time, therefore, randomization was unnecessary.<br>In PK studies in dogs, each group was examined individually, separated in time, and therefore, randomization was not needed.<br><br>For in vitro experiments (e.g. in multi-well plates), samples were allocated to different groups/treatments randomly.
Blinding	No blinding was performed.<br>In the studies with BDL rats, the investigators who performed BDL surgery, treatment administration, blood ammonia measurements, MRS measurements were not blinded to the treatment identity. However, ammonia measurements and MRS data analysis were performed by different investigators.<br><br>In the studies with DEN rats, the same investigators performed animal handling and dosing and they were not blinded to the treatment identity. For these studies, blinding was not relevant as the conclusions were made based on the objective data directly measured by a device (blood ammonia was measured by PocketChem device) and not processed afterwards.<br><br>In the PK studies, animal dosing and sampling was performed by one group of investigators, while plasma sample analysis was performed by another investigator. None of the investigators were blinded to the treatment identity.

Reporting for specific materials, systems and methods

We require information from authors about some types of materials, experimental systems and methods used in many studies. Here, indicate whether each material, system or method listed is relevant to your study. If you are not sure if a list item applies to your research, read the appropriate section before selecting a response.

Materials & experimental systems

n/a	Involved in the study	
☒	☐	Antibodies
☐	☒	Eukaryotic cell lines
☒	☐	Palaeontology and archaeology
☐	☒	Animals and other organisms
☒	☐	Clinical data
☒	☐	Dual use research of concern
☒	☐	Plants

Methods

n/a	Involved in the study	
☒	☐	ChIP-seq
☒	☐	Flow cytometry
☐	☒	MRI-based neuroimaging

Eukaryotic cell lines

Policy information about cell lines and Sex and Gender in Research

Cell line source(s)	Caco-2 cells were obtained from American Type Culture Collection.
Authentication	Cell line was not authenticated .
Mycoplasma contamination	Cell line was tested negative for mycoplasma contamination. The mycoplasma test was performed using the MycoAlert PLUS Mycoplasma Detection Kit (Lonza).
Commonly misidentified lines<br>(See ICLAC register)	No commonly misidentified cell lines were used in this study.

Animals and other research organisms

Policy information about studies involving animals; ARRIVE guidelines recommended for reporting animal research, and Sex and Gender in Research

Laboratory animals	In the PK studies, Beagle dogs (30-40 months old) were used. In the efficacy studies, Sprague Dawley (6-8 weeks old) and Wistar rats were used. Wistar rats were ordered based on their body weight, therefore, the age is not known.
Wild animals	The study did not involve wild animals

Reporting on sex	Only male animals were used in the in vivo studies, and sex was not considered in the study design.
Field-collected samples	The study did not involve samples collected from the field
Ethics oversight	Research in this manuscript complies with all relevant ethical regulations. Animal procedures in the studies with dogs were approved by the Comité Institutionnel de Protection des Animaux (CIPA) of the Institut National de la Recherche Scientifique (INRS) (Ethical Animal Protocol #2002-04). The INRS test facility is accredited by Association for Assessment and Accreditation of Laboratory Animal Care (AAALAC) and Canadian Council on Animal Care (CCAC). Animal experiments in rat model of N-nitrosodiethylamine (DEN)-induced liver injury were approved by the Institutional Animal Care and Use Committee (IUCAC) of Wuhan Servicebio Technology Co., Ltd. (protocol numbers: 2021071, 2021136, 2021137, 2022003). Animal experiments in bile duct ligated rats were conducted according to the Federal and local ethical guidelines, the protocols were approved by the local Committee on Animal Experimentation for the Canton de Vaud, Switzerland (VD3022.1).

Note that full information on the approval of the study protocol must also be provided in the manuscript.

Plants

Seed stocks	*Report on the source of all seed stocks or other plant material used. If applicable, state the seed stock centre and catalogue number. If plant specimens were collected from the field, describe the collection location, date and sampling procedures.*
Novel plant genotypes	*Describe the methods by which all novel plant genotypes were produced. This includes those generated by transgenic approaches, gene editing, chemical/radiation-based mutagenesis and hybridization. For transgenic lines, describe the transformation method, the number of independent lines analyzed and the generation upon which experiments were performed. For gene-edited lines, describe the editor used, the endogenous sequence targeted for editing, the targeting guide RNA sequence (if applicable) and how the editor was applied.*
Authentication	*Describe any authentication procedures for each seed stock used or novel genotype generated. Describe any experiments used to assess the effect of a mutation and, where applicable, how potential secondary effects (e.g. second site T-DNA insertions, mosiacism, off-target gene editing) were examined.*

Magnetic resonance imaging

Experimental design

Design type	Not applicable
Design specifications	Not applicable
Behavioral performance measures	None

Acquisition

Imaging type(s)	Magnetic Resonance Spectroscopy
Field strength	9.4T
Sequence & imaging parameters	SPECIAL sequence, TE=2.8 ms, TR=4000 ms
Area of acquisition	Cerebellum

Diffusion MRI ☐ Used ☒ Not used

Preprocessing

Preprocessing software	LCModel
Normalization	Water used as internal reference
Normalization template	Not applicable
Noise and artifact removal	Not applicable
Volume censoring	Not applicable

Statistical modeling & inference

Model type and settings	Not applicable
Effect(s) tested	Not applicable

Specify type of analysis: ☐ Whole brain ☐ ROI-based ☐ Both

Statistic type for inference (See Eklund et al. 2016)	Not applicable
Correction	Not applicable

Models & analysis

n/a	Involved in the study
☒	☐ Functional and/or effective connectivity
☒	☐ Graph analysis
☒	☐ Multivariate modeling or predictive analysis
